# Associations of Frailty Status and Sleep Quality With Incident Delirium: A Prospective Study in the UK Biobank

**DOI:** 10.1111/cns.70266

**Published:** 2025-02-24

**Authors:** Yueqing Huang, Guangqi Chen, Ning Ma, Li Wang, Yue Wang, Zhenyu Jia, Min Huang, Dingliu He

**Affiliations:** ^1^ Department of General Medicine The Affiliated Suzhou Hospital of Nanjing Medical University, Suzhou Municipal Hospital, Nanjing Medical University Suzhou Jiangsu China; ^2^ Department of General Medicine Suzhou Xiangcheng District Traditional Chinese Medicine Hospital Suzhou Jiangsu China; ^3^ School of Public Health Capital Medical University Beijing China; ^4^ Department of General Medicine Runda Community Health Service Center Suzhou Jiangsu China; ^5^ Department of General Medicine The Affiliated Zhangjiagang Hospital of Soochow University Suzhou Jiangsu China; ^6^ Department of Clinical Nutrition Yancheng Clinical College of Xuzhou Medical University, the First People's Hospital of Yancheng Yancheng Jiangsu China

**Keywords:** delirium incidence, frailty, interaction, prospective study, sleep quality

## Abstract

**Aims:**

Frailty and poor sleep are both risk factors for delirium, but their joint and interactive associations with incident delirium are unknown. The study aimed to examine the associations of frailty status and sleep quality with incident delirium.

**Methods:**

A total of 346,846 participants from the UK Biobank without delirium, dementia, or cognitive impairment at baseline were included. Incident delirium cases were identified through the International Classification of Diseases, Tenth Revision codes. A modified‐version Fried phenotype was utilized to evaluate frailty status. A sleep score comprising five sleep characteristics was used to quantify sleep quality. Cox proportional hazard models were applied to calculate the hazard ratios (HRs) and 95% confidence intervals (CIs) for the associations. Also, their interactions on delirium incidence on the multiplicative and additive scales were quantified.

**Results:**

During a median follow‐up of 13.8 years, 6554 (1.9%) cases of incident delirium were documented. Prefrail and frail individuals had a 34% (HR = 1.34; 95% CI: 1.27–1.41) and 98% (HR = 1.98; 95% CI: 1.78–2.19) increased risk of delirium, respectively, compared with nonfrail controls. Each 1‐point increment in frailty score was associated with a 25% (HR = 1.25; 95% CI: 1.21–1.28) increased risk of delirium. Healthy sleep significantly counteracted the increased risk of delirium due to frailty (*P* for multiplicative interaction = 0.026). In the poor and healthy sleep stratification, the corresponding HRs (95% CIs) were 2.07 (1.86–2.31) and 1.28 (0.83–1.97), respectively. Moreover, we observed the highest delirium risk in frail individuals with poor sleep quality (HR = 2.08; 95% CI: 1.83–2.35), with evidence of an additive interaction.

**Conclusions:**

Both physical frailty and poor sleep are significantly associated with higher delirium risk. Achieving healthy sleep could not only lower the risk of delirium but also greatly offset the adverse impact of frailty on delirium.

## Graphical abstract

The study observed that both physical frailty and poor sleep were significantly and linearly associated with a higher risk of incident delirium. Achieving healthy sleep could not only lower the risk of delirium but also greatly offset the adverse impact of frailty on delirium.

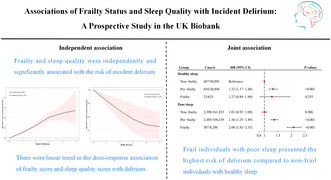

## 1 Introduction

Delirium, an acute‐onset neurocognitive syndrome, is often marked by inattention, disorganized thinking, and significant fluctuations in consciousness [1]. The prevalence of delirium escalates considerably with age and is commonly prevalent in hospitalized patients, affecting up to half of this population [1, 2]. In addition to raising the risk of stroke, dementia, and mortality, delirium affects the prognosis of hospitalized patients, resulting in considerable economic and social costs [1, 2]. Despite its potential reversibility, effective interventions or medications for delirium are currently unavailable [3]. Therefore, identifying modifiable contributing factors is urgently required.

Physical frailty, a significant global public health issue related to aging, is marked by diminished multisystem functioning and heightened vulnerability to stressors [4], resulting in an elevated risk of adverse outcomes including disability, hospitalization, and mortality [5]. Despite the heightened focus on frailty in the context of neurological disorders such as stroke [6], dementia [7], and Parkinson's disease [8], research on delirium is still scarce. The majority of studies investigating the link between frailty and delirium have been limited to postoperative patients [9, 10, 11]. A meta‐analysis of 15 cohort studies concluded that frailty may increase the risk of postoperative delirium (POD) [9]. While postoperative patients experience a higher prevalence of delirium, nonsurgical hospitalized patients are also at risk [1]. Delirium in nonsurgical patients is influenced by a broader range of factors, such as chronic comorbidities, acute medical conditions, infections, and medications, differing substantially from the surgical stressors observed in POD [2]. Investigating delirium in the broader hospitalized population, rather than focusing solely on surgical patients, provides a more comprehensive understanding of this multifactorial syndrome and its associated risk factors [2]. However, the few studies available exploring the relationship in these nonsurgical patients were constrained by cross‐sectional designs and small sample sizes [12, 13, 14]. Advanced age, low socioeconomic status (SES), functional impairment, and psychiatric comorbidities are shared features of frailty and delirium [1, 5]. Notably, earlier research has identified specific frailty components linked to delirium, such as decreased grip strength (GS) [15], slow gait speed [16], weight loss [17], exhaustion [18], and low physical activity (PA) [19]. Also, the close connection of cognitive decline (a key feature of delirium) with frailty has been widely documented [20]. Elevated levels of inflammatory markers, atherosclerosis, and chronic malnutrition caused by deficiencies in multiple macronutrients might represent potential mechanisms underlying the association between frailty and the risk of delirium [21]. Despite this, to our knowledge, no prospective research has systematically addressed the long‐term association between frailty status and the risk of incident delirium. Furthermore, sustaining a healthy lifestyle (e.g., healthy sleep) has been suggested to reduce the risk of frailty [22].

Poor sleep is widely acknowledged as a predisposing factor for delirium [23]. Yet, the majority of studies examining this association have centered on POD [24, 25]. Furthermore, prior investigations have generally focused on individual sleep behaviors [26, 27], ignoring the interconnected nature of these behaviors. Notably, a composite sleep score reflecting sleep quality that integrates five behaviors (sleep duration, insomnia, daytime sleepiness, snoring, and chronotype) has been validated to be significantly associated with several neurological disorders [28, 29], but it has not yet been linked to delirium. Prior research did report an association between poor sleep burden and delirium [30]; however, it included only four of the specified sleep behaviors, excluding snoring, and did not consider the role of frailty. Frailty status and sleep quality could be interrelated, jointly affecting health. For instance, longer sleep duration could reduce time for PA, increasing the likelihood of frailty. Significantly, an earlier study has suggested that adopting healthy sleep could alleviate the progression of frailty [31]. Hence, it is plausible to propose that healthy sleep could lessen the harmful influence of frailty on delirium risk. Nonetheless, the possible combined and interactive associations of both factors with delirium incidence remain uncertain.

Based on the knowledge gaps described above, we first hypothesized that frailty status and sleep quality are independently associated with the risk of delirium. Second, we hypothesized that maintaining healthy sleep would mitigate the risk of delirium associated with frailty. Leveraging data from the UK Biobank, we executed a prospective analysis to assess the independent, joint, and interactive associations of frailty status and sleep quality with the risk of incident delirium.

## 2 Methods

### 2.1 Study Participants

The UK Biobank, a vast prospective cohort with ongoing follow‐up, provided the participants for our study. It enrolled over 500,000 middle‐aged and older adults across 22 assessment centers in the UK at baseline between 2006 and 2010. Medical information on the participants was collected via touchscreen questionnaires and physical and biological examinations. The cohort has been described in detail in early research [32]. The ethical approval for the UK Biobank project was granted by the Northwest Multi‐Centre Research Ethics Committee (21/NW/0157). Informed consent was secured from all participants before recruitment. The present study was conducted using the UK Biobank resource under Application Number 104283. A total of 502,366 participants contributed data to the study. Individuals without complete information for frailty status (*n* = 34,639) or sleep quality (*n* = 76,922), those with delirium, dementia, or cognitive impairment at baseline (*n* = 145), and those without any hospital admission after baseline survey (*n* = 43,814) were excluded. Ultimately, 346,846 participants were included in the current analysis (Figure S1).

### 2.2 Exposures

We assessed frailty status using a revised frailty phenotype tailored to the UK Biobank data [33], consisting of five components: weight loss, exhaustion, low PA, slow gait speed, and low GS. Each component was assigned a score of 1 if the criteria were met and 0 if not, yielding a continuous frailty score from 0 to 5, where higher scores denote greater frailty. The definitions and scoring criteria for each component are detailed in Table S1. Following previous studies [7, 8, 33], individuals were classified as nonfrail (0 points), prefrail (1–2 points), and frail (≥ 3 points).

As in previous studies [34, 35], five sleep behaviors were integrated to assess sleep quality. Each sleep behavior was categorized as low or high risk, whereas low‐risk sleep behaviors included early chronotype, sleep 7–8 h/day, no frequent insomnia, no snoring, and no frequent sleepiness. Depending on the number of low‐risk sleep behaviors, participants received sleep scores ranging from 0 to 5, which were then categorized into three groups: low (0–1), intermediate (2–3), and high (4–5) sleep quality [34, 35]. The definitions and scoring criteria for each sleep behavior are displayed in Table S2.

### 2.3 Outcome

During the follow‐up period, the UK Biobank team accessed and released hospitalization dates and International Classification of Diseases, Tenth Revision (ICD‐10) codes for study participants from the National Health Service in the United Kingdom. Consistent with previous studies [18, 36], incident delirium cases were determined based on the first occurrence of ICD‐10 code F05 from hospitalization records during follow‐up. The follow‐up time was calculated as the time interval from the baseline recruitment date to the date of delirium incidence, death, or the censoring date (October 31, 2022, for England; August 31, 2022, for Scotland; and May 31, 2022 for Wales), whichever occurred first.

### 2.4 Covariates

The current study considered a range of potential covariates, including sociodemographic characteristics (age, sex, ethnicity, educational level, family income, employment status, and Townsend deprivation index [TDI]), lifestyle factors (smoking status, drinking status, PA, diet, and body mass index [BMI]), cognition, and medical histories (hypertension, diabetes, dyslipidemia, and cardiovascular diseases [CVD]). TDI is a composite variable reflecting the level of poverty in an area with higher scores being associated with higher levels of poverty. A healthy diet index including seven dietary components was employed to assess diet quality [37] (Table S3). Adequate PA was defined as undertaking vigorous PA of at least 75 min per week, moderate PA of at least 150 min per week, or equivalent combination [37]. Obesity was defined as BMI ≥ 30 kg/m^2^. The mean reaction time to correctly recognize card matches was used as a proxy for cognitive function [18]. Detailed measurements on covariates can be seen in Table S4. To maximize the sample size, we addressed the missing values in covariates by creating a unique category for categorical variables and by employing median interpolation for continuous variables.

### 2.5 Statistical Analyses

Descriptive analyses were conducted to summarize baseline characteristics of participants by delirium event, frailty, or sleep status at baseline. The normality of continuous variables was assessed using the Kolmogorov–Smirnov test. For variables following a normal distribution, data were expressed as mean (standard deviation, SD), and group comparisons were performed using *t*‐tests or ANOVA. For nonnormally distributed variables, data were expressed as median (interquartile range, IQR), and group comparisons were conducted using the Kruskal–Wallis test. Categorical variables were presented as frequency (%), and group comparisons were performed using the chi‐square test. Cox proportional hazard models were performed to calculate the hazard ratios (HRs) and 95% confidence intervals (CIs) for the associations of frailty status and sleep quality with delirium incidence. We tested the proportional hazard assumption with the Schoenfeld residual method and found no violations. Three Cox models with incrementally adjusted covariates were fitted. Model 1 adjusted for age and sex. Model 2 added ethnicity, educational level, family income, TDI, employment status, and lifestyle factors to Model 1. Model 3 incorporated cognition function, medical histories, and sleep quality or frailty status into Model 2.

A multistep analytic strategy was employed to investigate the associations. First, we processed the frailty score as a continuous variable to calculate the HRs for each 1‐point increase. Second, we used the nonfrailty group as a reference group to estimate the risk of incident delirium due to prefrailty and frailty. Also, the linear trends were tested by designating the frailty status as a continuous variable. Third, to explore the cumulative risk of increasing frailty scores, we used individuals scoring 0 as the referent and estimated HRs separately for those with scores between 1 and 5. Fourth, independent associations of the five frailty components with the risk of delirium adjusted for each other were also examined. The same analytic strategy was conducted for sleep quality. Moreover, the Kaplan–Meier (KM) method was adopted to map the cumulative hazards of frailty status or sleep quality on delirium incidence. The restricted cubic spline (RCS) model with 4 knots (percentiles of 5, 35, 65, and 95) and a reference value of 0 was employed to examine the dose–response relationship of frailty or sleep scores with incident delirium.

The joint associations of frailty status and sleep quality with the risk of delirium were examined using individuals categorized as nonfrailty and healthy sleep as the reference. Furthermore, the relationship between frailty status and delirium incidence was estimated at different stratifications of sleep quality. The interaction between frailty status and sleep quality was examined by incorporating a multiplicative interaction term in Model 3. The likelihood test was conducted to test the significance of the multiplicative interaction. Also, an additive interaction model was constructed and assessed using the relative excess risk due to interaction (RERI) and the attributable proportion due to interaction (AP) [38]. If the CIs for RERI and AP did not include 0, a significant additive interaction was suggested [38]. Given the markedly skewed distribution of sleep quality, we dichotomized sleep quality into poor (0–4 scores) and healthy (5 scores) in joint, stratified, and interaction analyses for ease of interpretation and to ensure a sufficient number of outcome events for statistical power.

Several additional analyses were performed to examine the robustness of our findings. First, to mitigate reverse causation bias, we eliminated participants with delirium cases that occurred within the first 2 years of follow‐up. Second, given the potential bias of missing data for covariates, multiple imputation techniques based on chained equations were implemented. Third, we reanalyzed the associations after excluding cases described as “Delirium superimposed on dementia” (ICD 10 code: F05.1) at follow‐up [36]. Fourth, we considered individuals who underwent surgery within 3 days prior to the incidence of delirium as POD and repeated the main analysis with this as the outcome [36]. Fifth, we rerun the association analyses after excluding individuals with missing data in covariates. Sixth, after further adjustment for baseline cancer, the association analyses were repeated. Seventh, to further explore the temporal relationship between frailty and delirium, stratified analyses were performed based on follow‐up duration (baseline to 5 years vs. 5–15 years). Eighth, since participants with CVD might affect the study outcome, sensitivity analyses excluding these baseline patients were implemented. Ninth, dementia events occurring within the first 2 years of follow‐up were excluded to minimize potential reverse causation bias. Finally, subgroup analyses were performed to examine the potential modification role of sociodemographic and lifestyle factors on the frailty–delirium association. All analyses were conducted with SAS (SAS Institute, Cary, NC, USA, version 9.4) and R Statistical Software (version 4.0.2). Statistical significance was defined as a two‐tailed *p* value < 0.05.

## 3 Results

### 3.1 Baseline Characteristics

Baseline characteristics of the study participants are presented in Table 1. During the 13.81‐year median follow‐up, 6554 (1.9%) cases of incident delirium were recorded. Of 346,846 participants (mean age [SD], 56.85 [8.05] years; 55.30% female), 8905 (2.57%) were classified as frail, while 8845 (2.55%) were noted to have low sleep quality. Compared to the nondelirium group, the delirium group was older, more likely to be male, White, current smokers, frequent drinkers, and unemployed. They also had lower levels of education and income, greater social deprivation, unhealthy diets, inadequate PA, and longer reaction times. Obesity, low‐quality sleep, frailty, hypertension, diabetes, dyslipidemia, and CVDs were more prevalent among patients with delirium (all *p* < 0.001). Similar findings were obtained when describing baseline characteristics according to frailty status or sleep quality (Tables S5 and S6).

**TABLE 1:** Baseline characteristics of included participants.

Characteristic	Overall	Incidence of delirium		*p*
		No	Yes	
*n*	346,846	340,292	6554	
Age, years (mean [SD])	56.85 (8.05)	56.72 (8.04)	63.55 (5.24)	< 0.001
Sex				
Female	191,812 (55.30)	189,001 (55.54)	2811 (42.89)	< 0.001
Male	155,034 (44.70)	151,291 (44.46)	3743 (57.11)	
Ethnicity				
White	317,120 (91.43)	311,070 (91.41)	6050 (92.31)	< 0.001
Mixed	12,290 (3.54)	12,021 (3.53)	269 (4.10)	
Asian	11,427 (3.29)	11,278 (3.31)	149 (2.27)	
Black	1682 (0.48)	1661 (0.49)	21 (0.32)	
Chinese	907 (0.26)	902 (0.27)	5 (0.08)	
Others	3420 (0.99)	3360 (0.99)	60 (0.92)	
Educational level				
Less than college	233,883 (67.43)	228,801 (67.24)	5082 (77.54)	< 0.001
Equal to or more than college	110,304 (31.80)	108,917 (32.01)	1387 (21.16)	
Unknow/missing value	2659 (0.77)	2574 (0.76)	85 (1.30)	
Townsend deprivation index (median [interquartile range])	−2.24 (−3.69, 0.31)	−2.24 (−3.69, 0.29)	−1.76 (−3.45, 1.37)	< 0.001
Family income, £				
< 18,000	65,432 (18.86)	630,98 (18.54)	2334 (35.61)	< 0.001
18,000–51,999	157,900 (45.52)	155,380 (45.66)	2520 (38.45)	
> 52,000	77,987 (22.48)	77,458 (22.76)	529 (8.07)	
Unknow/missing value	45,527 (13.13)	44,356 (13.03)	1171 (17.87)	
Employment status				
Current employed	196,706 (56.71)	195,086 (57.33)	1620 (24.72)	< 0.001
Current no employed	149,233 (43.03)	144,316 (42.41)	4917 (75.02)	
Unknow/missing value	907 (0.26)	890 (0.26)	17 (0.26)	
Smoking status				
Never smoking	185,243 (53.41)	182,553 (53.65)	2690 (41.04)	< 0.001
Ever smoking	124,409 (35.87)	121,543 (35.72)	2866 (43.73)	
Current smoking	36,250 (10.45)	35,282 (10.37)	968 (14.77)	
Unknow/missing value	944 (0.27)	914 (0.27)	30 (0.46)	
Drinking status				
Never drinking	26,298 (7.58)	25,479 (7.49)	819 (12.50)	< 0.001
Special occasions only	38,753 (11.17)	37,883 (11.13)	870 (13.27)	
1–3 times a month	38,434 (11.08)	37,795 (11.11)	639 (9.75)	
Once or twice a week	90,050 (25.96)	88,568 (26.03)	1482 (22.61)	
3 or 4 times a week	80,885 (23.32)	79,654 (23.41)	1231 (18.78)	
Daily or almost daily	72,277 (20.84)	70,769 (20.80)	1508 (23.01)	
Unknow/missing value	200 (0.06)	193 (0.06)	7 (0.11)	
Healthy diet index, (mean [SD])	3.32 (1.32)	3.32 (1.32)	3.27 (1.36)	< 0.001
Obesity				
No	260,347 (75.06)	255,933 (75.21)	4414 (67.35)	< 0.001
Yes	86,499 (24.94)	84,359 (24.79)	2140 (32.65)	
Adequate physical activity				
No	89,893 (25.92)	87,951 (25.85)	1942 (29.63)	< 0.001
Yes	251,732 (72.58)	247,307 (72.67)	4425 (67.52)	
Unknow/missing value	5221 (1.51)	5034 (1.48)	187 (2.85)	
Reaction time, ms (mean [SD])	558.41 (114.90)	557.55 (114.30)	603.36 (135.20)	< 0.001
Baseline disease status				
Cardiovascular disease	28,195 (8.13)	26,837 (7.89)	1358 (20.72)	< 0.001
Diabetes	21,808 (6.29)	20,650 (6.07)	1158 (17.67)	< 0.001
Hypertension	194,554 (56.09)	189,534 (55.70)	5020 (76.59)	< 0.001
Dyslipidemia	163,120 (47.03)	159,075 (46.75)	4045 (61.72)	< 0.001
Sleep quality				
Low	8845 (2.55)	8598 (2.53)	247 (3.77)	< 0.001
Intermediate	139,041 (40.09)	136,125 (40.00)	2916 (44.49)	
High	198,960 (57.36)	195,569 (57.47)	3391 (51.74)	
Frailty status				
Nonfrailty	212,828 (61.36)	209,743 (61.64)	3085 (47.07)	< 0.001
Prefrailty	125,113 (36.07)	122,174 (35.90)	2939 (44.84)	
Frailty	8905 (2.57)	8375 (2.46)	530 (8.09)	

*Note:* Data were presented as mean (standard deviation) for continue variables with normal distribution, median (interquartile range) for continue variables with nonnormal distribution, and frequency (%) for categorized variables.

### 3.2 Independent Associations Between Frailty Status and Risk of Delirium

**FIGURE 1:**
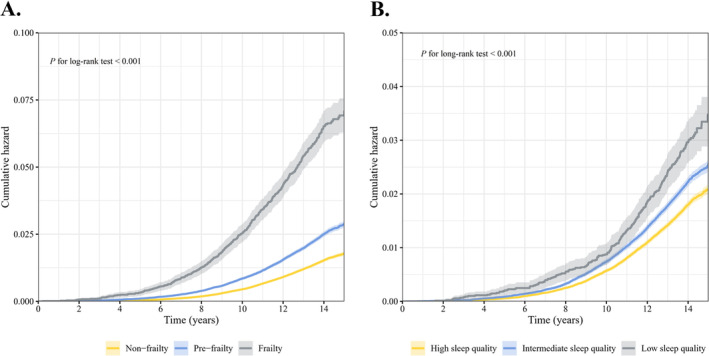
Cumulative incidence of delirium according to frailty status (A) and sleep quality (B) at baseline.

During follow‐up, frail individuals had the highest cumulative risk of developing delirium, followed by prefrail and nonfrail individuals (Figure 1A). The findings of the RCS analyses prompted no evidence of nonlinear relationships between frailty scores and the incidence of delirium (nonlinear *p* = 0.205, Figure S2A). Compared to participants with a frailty score of 0, the fully adjusted HRs (95% CI) for those with scores between 1 and 5 were 1.26 (1.19–1.34), 1.60 (1.48–1.74), 2.02 (1.80–2.25), 2.06 (1.68–2.52), and 2.60 (1.53–4.41), respectively, demonstrating a clear linear trend (Table 2). Each 1‐point increase in frailty score was associated with a 25% (21%–28%) increased risk of delirium (Table 2). Also, we found that prefrail individuals had a 34% increased risk of delirium (HR: 1.34; 95% CI: 1.27–1.41) compared to nonfrail individuals, and frail individuals had a 98% heightened risk (HR: 1.98; 95% CI: 1.78–2.19) (*P* for trend < 0.001, Table 2).

**TABLE 2:** Association of frailty status at baseline with risk of incident delirium.

Exposures	Case/*n*	Person‐years	HRs (95% CIs)					
			Model 1	*p*	Model 2	*p*	Model 3	*p*
Frailty								
Nonfrailty	3085/212,828	2,936,759	1.00 (Reference)		1.00 (Reference)		1.00 (Reference)	
Prefrailty	2939/125,113	1,718,751	1.64 (1.56–1.72)	< 0.001	1.42 (1.35–1.50)	< 0.001	1.34 (1.27–1.41)	< 0.001
Frailty	530/8905	121,243	3.67 (3.35–4.03)	< 0.001	2.38 (2.15–2.63)	< 0.001	1.98 (1.78–2.19)	< 0.001
*p* for trend			< 0.001		< 0.001		< 0.001	
Frailty score								
0	3085/212,828	2,936,759	1.00 (Reference)		1.00 (Reference)		1.00 (Reference)	
1	2020/98,181	1,349,987	1.45 (1.37–1.54)	< 0.001	1.32 (1.24–1.40)	< 0.001	1.26 (1.19–1.34)	< 0.001
2	919/26,932	368,764	2.28 (2.12–2.45)	< 0.001	1.79 (1.65–1.93)	< 0.001	1.60 (1.48–1.74)	< 0.001
3	412/7292	99,405	3.54 (3.19–3.92)	< 0.001	2.40 (2.15–2.68)	< 0.001	2.02 (1.80–2.25)	< 0.001
4	104/1482	20,071	4.15 (3.41–5.05)	< 0.001	2.59 (2.11–3.16)	< 0.001	2.06 (1.68–2.52)	< 0.001
5	14/131	1766	5.34 (3.16–9.04)	< 0.001	3.18 (1.87–5.39)	< 0.001	2.60 (1.53–4.41)	< 0.001
Per 1‐score increase			1.49 (1.45–1.52)	< 0.001	1.32 (1.28–1.35)	< 0.001	1.25 (1.21–1.28)	< 0.001

*Note:* Model 1 adjusted for age and sex; Model 2 additionally adjusted for ethnicity, educational level, family income, TDI, employment status, smoking, drinking, obesity, healthy diet score, and physical activity; and Model 3 additionally adjusted for cardiovascular diseases, diabetes, hypertension, dyslipidemia, reaction time, and sleep quality.

Abbreviations: CI, confidence interval; HR, hazard ratio.

After adjusting the covariates in Model 3 and mutually adjusting the five frailty components, we observed that all five components were associated with the incidence of delirium to varying degrees, with slow gait speed exhibiting the strongest association (Table S7). The HRs (95% CI) were 1.15 (1.08–1.23) for weight loss; 1.22 (1.13–1.30) for exhaustion; 1.45 (1.35–1.55) for slow gait speed; 1.21 (1.12–1.31) for low GS; and 1.19 (1.10–1.28) for low PA.

### 3.3 Independent Associations Between Sleep Quality and Risk of Delirium

The highest cumulative incidence was observed in individuals with low sleep quality (Figure 1B). The RCS analysis did not reveal a nonlinear relationship between sleep scores and incident delirium (nonlinear *p* = 0.169, Figure S2B). Compared to individuals with a sleep score of 5, the fully adjusted HRs (95% CI) for those with scores between 0 and 4 were 1.65 (1.12–2.42), 1.25 (1.08–1.44), 1.15 (1.05–1.25), 1.08 (1.01–1.16), and 1.01 (0.94–1.09), respectively (Table S8). Using individuals with high sleep quality as the reference group, we observed that low sleep quality increased the risk of delirium by 15% (HR = 1.15; 95% CI: 1.01–1.31) (*P* for trend < 0.001, Table S8). Regarding the individual components of sleep quality, we found that healthy sleep duration and no frequent daytime sleepiness were significant protective factors (Table S9).

### 3.4 Joint and Interaction Associations of Frailty and Sleep With Incident Delirium

**FIGURE 2:**
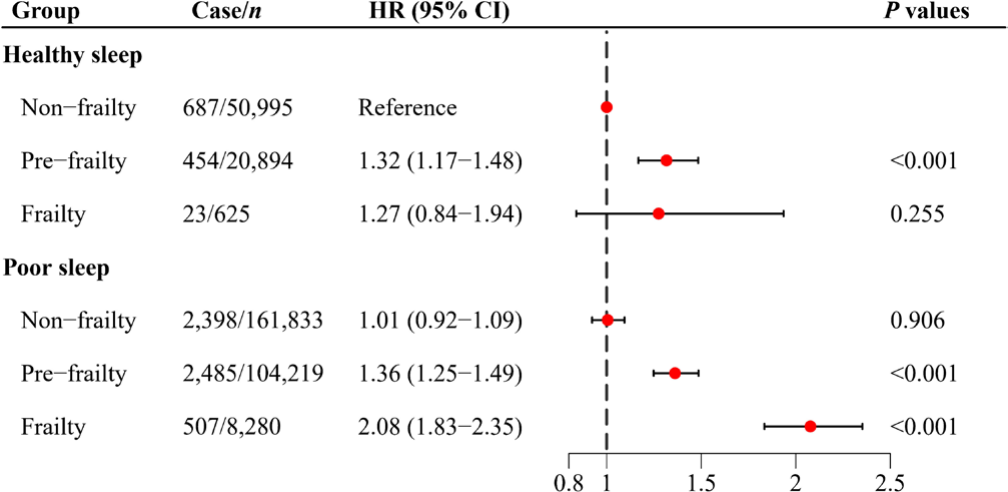
Joint associations of frailty and sleep quality with risk of delirium. Model adjusted for age, sex, ethnicity, educational level, family income, Townsend deprivation index, employment status, smoking status, drinking status, obesity, healthy diet scores, physical activity, cardiovascular diseases, diabetes, hypertension, dyslipidemia, and reaction time. CI, confidence interval; HR, hazards ratio.

The joint associations of frailty and sleep on delirium risk are displayed in Figure 2. Frail individuals with poor sleep presented the highest risk of delirium compared to nonfrail individuals with healthy sleep (HR = 2.08; 95% CI: 1.83–2.35). Additionally, we found that healthy sleep modified the association of frailty with risk of delirium, with evidence of a multiplicative interaction (*P* for multiplicative interaction = 0.026) (Table 3). In the group with poor sleep, frailty significantly increased the risk of delirium (HR = 2.07; 95% CI: 1.86–2.31). However, in the healthy sleep group, the association between frailty and risk of delirium was substantially weakened and not statistically significant (HR = 1.28; 95% CI: 0.83–1.97). The significant RERI indicated a positive additive interaction between sleep and frailty on delirium incidence (Table 3). Among frail individuals with unhealthy sleep, the RERI was 0.798 (95% CI: 0.243–1.352), suggesting a 0.798 relative excess risk due to the additive interaction.

**TABLE 3:** Stratification and interactive associations of frailty status and sleep quality with the risk of incident delirium.

Frailty status	Sleep quality^a^		Additive interaction^b^		*P* for multiplicative interaction^b^
	Poor	Healthy	RERI (95% CI)	AP (95% CI)	
Nonfrailty	1.00 (Reference)	1.00 (Reference)	0.798 (0.243, 1.352)	0.384 (0.124, 0.644)	0.026
Prefrailty	1.35 (1.28–1.44)	1.32 (1.16–1.49)			
Frailty	2.07 (1.86–2.31)	1.28 (0.83–1.97)			

Abbreviations: CI, confidence interval; HR, hazards ratio; RERI, relative excess risk due to interaction.

^a^ All results were adjusted for age, sex, ethnicity, educational level, family income, Townsend deprivation index, employment status, smoking status, drinking status, obesity, healthy diet scores, physical activity, cardiovascular diseases, diabetes, hypertension, dyslipidemia, and reaction time.

^b^ The multiplicative and additive interaction was evaluated between sleep quality (healthy vs. poor) and frailty status (non‐frailty vs. frailty). A significantly additive interaction was indicated if 95% CI of RERI did not contain 0 and 95% of AP did not contain one.

### 3.5 Sensitivity and Subgroup Analyses

The associations of frailty status with the risk of incident delirium were generally consistent with the primary analysis in the following sensitivity analyses: (1) eliminating individuals with delirium cases within the first 2 years of follow‐up (Table S10); (2) imputing missing data on covariates with multiple imputation techniques (Table S11); (3) excluding cases of dementia‐related delirium (Table S12); (4) using only POD as the study outcome (Table S13); (5) excluding individuals with missing values in covariates (Table S14); (6) adjusting for baseline cancer (Table S15); (7) stratifying by follow‐up period (Table S16); (8) excluding patients with CVD at baseline (Table S17); and (9) excluding individuals occurring delirium or all‐cause dementia within the first 2 years of follow‐up (Table S18). The association of frailty with delirium risk was generally similar across subgroups except for gender and age subgroups (all *P* for interaction > 0.05) (Table S19). Specifically, the detrimental impact of frailty on delirium was more pronounced in older participants and females (*P* for interaction < 0.01) compared to younger adults and males (Table S19).

## 4 Discussion

Building on a prospective cohort of 346,846 individuals, this study is the first to investigate the independent, joint, and interactive associations of frailty status and sleep quality with the risk of delirium. Compared with nonfrail individuals, those with prefrail and frail substantially raised the risk of delirium by 34% and 98%, respectively. Achieving healthy sleep was associated with a markedly reduced risk of delirium. The risk of delirium in frail individuals with poor sleep was 2.08 times higher than in nonfrail individuals with healthy sleep. Healthy sleep facilitated a significant reduction in the harmful association between frailty and delirium risk. Additionally, the study provided first‐hand evidence that frailty and poor sleep produce a statistically significant interaction on the incidence of delirium.

To our knowledge, the present study is the first to investigate the association between frailty status and future risk of delirium in a large prospective cohort study with a median follow‐up of over 13 years. Our findings reveal that frailty significantly augmented the risk of delirium, which is consistent with the findings of previous studies [9, 10, 11]. For example, a large meta‐analysis of 15 cohort studies involving 3250 surgical patients confirmed that frailty was significantly associated with an increased risk of POD [9]. A retrospective study by Cheng et al. [10] in 2080 cardiac surgery patients discovered that frail patients were more likely to develop delirium. Unfortunately, these studies are potentially limited to vulnerable populations with small samples (i.e., postoperative patients with specific diseases) [9, 10, 11]. Although postoperative patients face a higher incidence of delirium, nonsurgical individuals are equally susceptible to delirium [1]. However, the few existing studies investigating the relationship in these nonsurgical individuals suffered from limitations in cross‐sectional design and small sample sizes [12, 13, 14]. Using data from 89 older hospitalized patients, Bellelli et al. [12] conducted a cohort study that observed a higher incidence of delirium in frail patients compared to their counterparts. Our findings complement and extend previous evidence that frailty might be independently associated with the incidence of delirium, regardless of whether the individuals have undergone surgical treatment or not. Moreover, we observed that the hazard of frailty on delirium was more evident in women and older adults, possibly because these disadvantaged groups are more susceptible to frailty, which increases the risk of adverse outcomes [5]. Given the reversibility of frailty and the relatively low cost of measurement [4], early screening to identify and ameliorate those who are prefrail or frail is of paramount relevance to delirium prevention, especially among vulnerable populations such as women and the elderly.

Although the specific mechanisms by which frailty increases the risk of future delirium have not been fully elucidated, increased levels of inflammatory factors, atherosclerosis, and chronic malnutrition due to multiple macronutrient deficiencies might be potential mechanisms [21]. For instance, frailty is recognized as an indicator of elevated levels of inflammatory factors (e.g., C‐reactive protein, interleukins, etc.) [5], which have also been implicated as triggers for the incidence of delirium [23]. Significantly, the study revealed that all five components (i.e., weight loss, exhaustion, slow gait speed, low GS, and low PA) of frailty were independent risk factors for the incidence of delirium. Compared with the other components, we found that slow walking speed was most related to the risk of delirium, which is consistent with previous findings [16]. A 1‐year prospective study in 215 patients undergoing urologic surgery demonstrated that slow walking speed was an independent risk factor for POD [16]. Supporting our finding regarding weight loss, a retrospective study based on intensive care unit patients indicated that underweight patients had a significantly higher incidence of delirium than normal‐weight patients [17]. Similar to our findings for exhaustion, a prospective cohort study revealed the strongest correlation between tiredness and the incidence of delirium among the four depressive symptoms considered [18]. Dopamine imbalance has been proposed as a possible neurobiological mechanism for the link between fatigue and delirium [8, 23]. Besides, low GS and PA have been documented as significant risk factors for delirium [15, 19]. Generally, existing research on the relationship between frailty components and delirium is rather limited. Our findings augment and broaden previous knowledge, emphasizing that interventions targeting any of the frailty components could lower the risk of delirium.

In addition to frailty, sleep disorders are well recognized as another modifiable risk factor for delirium [23]. However, most current published studies remain to be centered on POD in the special population with small samples [24, 25, 26, 27]. For example, a review that included 12 studies with sample sizes ranging from 40 to 432 confirmed that sleep disorders may be associated with POD [24]. Furthermore, most of the extant studies on this topic assessed sleep disorders by only one sleep behavior or did not specify the type of sleep disorder [26, 27]. A retrospective cohort study of 489 patients undergoing surgery reported that sleep disorders assessed by insomnia were associated with a higher incidence of POD [26]. Nevertheless, sleep behaviors are generally interrelated, meaning that changes in one sleep behavior tend to trigger modifications in other behaviors. Therefore, when evaluating sleep health, it is crucial to jointly consider multiple sleep behaviors. The present study extended previous work by for the first time demonstrating that sleep quality integrating five sleep behaviors (snoring, chronotype, sleep duration, insomnia, and sleepiness) was independently associated with delirium onset. Supporting our findings, a previous cohort study reported poor sleep burden measured by multiple sleep behaviors as a risk factor for delirium [30]. However, this study did not consider the role of snoring. Indeed, obstructive sleep apnea has been established as a nonnegligible risk factor for delirium [23, 24]. Notably, none of these studies considered the potential role of frailty when examining the relationship between sleep and delirium.

This study was the first to assess the joint and interactive associations of frailty status and sleep quality with the incidence of delirium. Specifically, the increased risk of delirium incidence caused by frailty was mitigated by healthy sleep and a statistically significant multiplicative interaction was observed, which was similar to previous findings [31, 34]. Zhu et al. [31] concluded in a prospective cohort study of 23,847 adults that maintaining a healthy sleep pattern lowered the risk of transitioning to frailty. Using data from the UK Biobank, Huang et al. [34] observed that healthy sleep decreased the elevated risk of mortality related to low levels of PA (a component of frailty). Also, we observed that individuals with both poor sleep and frailty faced the highest risk of delirium compared to their counterparts, stressing the imperative of considering both factors in delirium prevention. Similar to this finding, a recent cohort study reported that the combination of low PA (a frailty component) and poor sleep faced the highest risk of Parkinson disease [28]. Importantly, the significant additive interaction observed in the present study suggested that poor sleep and frailty synergistically increased the risk of delirium. About 79.8% of delirium risk was attributable to the interaction, meaning that the joint role of simultaneous exposure to poor sleep and frailty was much greater than the simple sum of their individual roles. The observed interaction between frailty and sleep quality could provide insight into the scientific basis for the formulation of public health policies for delirium prevention. Specifically, individuals who are considered frail at early screening should be more concerned about their sleep quality. Furthermore, well‐designed studies are required to validate our findings. The exact mechanisms underlying the interactions among sleep, frailty, and delirium are not fully understood. Poor sleep, such as short sleep duration and insomnia, has been associated with lower testosterone levels, increased oxidative stress, and elevated proinflammatory factors, which are known to heighten the risk of physical frailty [31, 39, 40]. Moreover, frailty itself is characterized by chronic low‐grade inflammation, impaired stress resilience, and reduced physiological reserve [4], all of which increase susceptibility to delirium [1, 2]. Healthy sleep, on the other hand, might counteract these processes by reducing neuroinflammation, enhancing immune regulation, and supporting overall physiological recovery, which in turn might mitigate the risk of frailty‐related delirium [31, 39, 40].

Several strengths of the study were noteworthy. The study was the first to examine the independent and joint associations of frailty status and sleep quality with the risk of delirium. The study's prospective design, large sample size, long follow‐up period, thorough adjustment for confounders, and multiple robust sensitivity analyses strengthened the reliability of our findings. Another major innovation of the study was that it provided first‐hand evidence that frailty status and sleep quality interact to contribute to the incidence of delirium. The interaction observed facilitated the implementation of precision prevention strategies specifically for at‐risk populations. Several limitations of this study should be acknowledged. First, despite efforts to adjust for various common confounders, the effect of residual confounders is an unavoidable limitation in observational studies. Second, the UK Biobank cohort had a low response rate (5.5%) and selection bias, as participants were predominantly healthier and less socioeconomically deprived compared to the general population [41], which might limit the generalizability of our findings to more diverse populations, particularly those with higher frailty burdens or from low‐income settings. However, previous studies have confirmed the generalizability of risk factor associations derived from the UK Biobank cohort [41]. Future studies should explore the observed associations in more diverse and high‐risk populations to validate our findings further. Third, while sensitivity analyses excluding the first 2 years of follow‐up events did not alter the robustness of the associations, the nature of observational studies still prevents us from inferring causality or ruling out reverse causality. Fourth, to reduce the potential impact of reverse causation bias, participants with baseline cognitive impairments or dementia were excluded from our primary analysis. While this approach helps to clarify the temporal relationship between frailty and incident delirium, it might have resulted in an underestimation of the associations, as individuals with cognitive impairments or dementia are at the highest risk for delirium. Fifth, the vast majority of the components of the exposures of interest in this study were obtained through participants' self‐reports, which may introduce information bias. However, previous studies by the UK Biobank have confirmed the validity of these measurements [33, 34]. Sixth, the reliance on hospitalization records for delirium diagnosis using ICD‐10 codes might lead to underreporting or misclassification, as delirium is often underdiagnosed in clinical settings, which might attenuate the strength of the observed associations. Nonetheless, the use of ICD‐10 codes ensures a standardized diagnostic approach, and the large sample size minimizes the impact of potential misclassification bias. Seventh, although both frailty status and sleep inherently change over time, data unavailability made it impossible to investigate the impact of their longitudinal changes on outcomes. Future studies are merited to utilize their repeated‐measures data for research and analysis. Finally, given that the study's participants were predominantly middle‐aged and older Whites, the generalizability of the findings might be limited.

## 5 Conclusion

In this prospective cohort study with a median follow‐up period of over 13 years, we revealed that frailty status and sleep quality were significantly and independently associated with delirium incidence. Moreover, our analyses indicated that healthy sleep significantly lessened the harmful impact of frailty on delirium incidence, supported by evidence of a multiplicative interaction. Significantly, we found a substantially increased risk of delirium in frail individuals with poor sleep, and additive interactions between frailty and sleep. Our findings stressed the imperative of incorporating early screening for frailty and sleep into the primary prevention of delirium.

## Author Contributions

Y.H., G.C., Z.J., M.H., and D.H. conceived and designed the study. Y.H., G.C., and N.M. prepared the data. Y.H., G.C., and N.M. conducted the data analysis, performed interpretation of the results, and drafted the paper. N.M., L.W., Y.W., Z.J., M.H., and D.H. made critical revisions. All authors agreed on the final version of the paper and took responsibility for its content.

## Ethics Statement

The Northwest Multi‐Center Research Ethics Committee provided ethical approval for the UK Biobank project.

## Consent

All participants gave informed consent before being recruited.

## Conflicts of Interest

The authors declare no conflicts of interest.

## Supporting information


Data S1.

## Data Availability

Data supporting the findings of this study from the UK Biobank team. The UK Biobank data are available on application (www.ukbiobank.ac.uk/). The analytic methods and study materials that support the findings of this study will be available from the corresponding author upon request.
