# Supplementary material for: Associations of Frailty Status and Sleep Quality With Incident Delirium: A Prospective Study in the UK Biobank
Source: CNS Neurosci Ther. 2025 Feb 24;31(2):e70266. doi: 10.1111/cns.70266 (PMC11848733; doi:10.1111/cns.70266)
Supplement: Supplementary file 1 — Data S1. [file CNS-31-e70266-s001.docx]

| **Table S1** Definitions and thresholds for frailty components in the UK Biobank | | |
| --- | --- | --- |
| **Individual components** | **Criteria** | **Field IDs** |
| **Weight loss** | “Compared with one year ago, has your weight changed?”  Response:   - Yes, lost weight = 1; - Other = 0; - Prefer not to answer = missing data. | 2306 |
| **Exhaustion** | “Over the past two weeks, how often have you felt tired or had little energy?”  Response:   - More than half the days or nearly every day = 1; - Other = 0; - Prefer not to answer = missing data. | 2080 |
| **Low physical activity** | “In the last 4 weeks did you spend any time doing the following? (You can select more than one answer)”  Response:   - Walking for pleasure (not as a means of transport) = 0; - Other exercises (eg: swimming, cycling, keep fit, bowling) = 0; - Strenuous sports = 0; - Light DIY (eg: pruning, watering the lawn): - Frequency of once per week or less=1; - Frequency of more than once per week=0; - Heavy DIY (eg: weeding, lawn mowing, carpentry, digging) = 0; - None of the above = 1; - Prefer not to answer = missing data. | 6164, 1011 |
| **Slow gait speed** | “How would you describe your usual walking pace?”  Response:   - Slow = 1; - Other = 0; - Prefer not to answer = missing data. | 924 |
| **Low grip strength** | Measured grip strength expressed in kg by sex- and BMI- adjusted cut-off points.  Cut-off points:  Men   - If BMI ≤24.0 kg/m^2^ & grip strength ≤29 kg - If BMI 24.1 to 28.0 kg/m^2^ & grip strength ≤30 kg - If BMI >28.0 kg/m^2^ & grip strength ≤32 kg   Women   - If BMI ≤23.0 kg/m^2^ & grip strength ≤17 kg - If BMI 23.1 to 26.0 kg/m^2^ & grip strength ≤17.3 kg - If BMI 26.1 to 29.0 kg/m^2^ & grip strength ≤18 kg - If BMI >29.0 kg/m^2^ & grip strength ≤21 kg - If data on BMI or grip strength is not available = missing data. | 31, 21001, 46, 47 |

Abbreviations: BMI, body mass index.

| **Table S2** Definitions and cut-off values for healthy sleep behaviors in the UK Biobank | | |
| --- | --- | --- |
| **Healthy behavior** | **Criteria** | **Field IDs** |
| Early chronotype | “Do you consider yourself to be?”  Response:   - Definitely a 'morning' person/ More a 'morning' than 'evening' person = 1; - More an 'evening' than a 'morning' person/Definitely an 'evening' person = 0; - Prefer not to answer/Do not know = missing data. | 1180 |
| Sleep 7-8 h/day | “About how many hours sleep do you get in every 24 hours? (please include naps)”  Response:   - 7-8 h/day = 1; - Other = 0; - Prefer not to answer/Do not know = missing data. | 1160 |
| No frequent insomnia | “Do you have trouble falling asleep at night or do you wake up in the middle of the night?”  Response:   - Never/rarely/sometimes =1; - Usually = 0; - Prefer not to answer = missing data. | 1200 |
| No snoring | “Does your partner or a close relative or friend complain about your snoring?”  Response:   - No = 1; - Yes = 0; - Prefer not to answer = missing data. | 1210 |
| No frequent daytime sleepiness | “How likely are you to doze off or fall asleep during the daytime when you don't mean to? (e.g. when working, reading or driving)”  Response:   - Never/rarely/sometimes =1; - Often/All of the time= 0; - Prefer not to answer/Do not know = missing data. | 1220 |

| **Table S3.** Measures of covariates at baseline in the UK Biobank | | | | |
| --- | --- | --- | --- | --- |
| **Variable** | **Filed ID** | **Question or description** | **Categories from raw data** | **Categories for the current study** |
| Ethnicity | 21000 | Ethnic background | White;  Mixed;  Asian or Asian British;  Black or Black British;  Chinese;  Other ethnic group. | 1 = White;  2 = Mixed;  3 = Asian;  4 = Black;  5 = Chinese;  6 = Others. |
| Education | 6138 | Qualifications | College or University degree;  A levels/AS levels or equivalent;  O levels/GCSEs or equivalent;  CSEs or equivalent;  NVQ or HND or HNC or equivalent;  Other professional qualifications eg: nursing, teaching. | 0 = College or university degree;  1 = Others;  9 = Unknow or missing value. |
| Family income | 738 | Average total household income before tax (unit: £) | Less than 18,000;  18,000 to 30,999;  31,000 to 51,999;  52,000 to 100,000;  Greater than 100,000; | 1 = <18,000;  2 = 18,000 to 51,999;  3 = >52,000;  9 = Unknow or missing value. |
| Employment | 6142 | Current employment status | In paid employment or self-employed;  Retired;  Looking after home and/or family;  Unable to work because of sickness or disability;  Unemployed;  Doing unpaid or voluntary work;  Full or part-time student;  None of the above. | 0 = Employed (including those paid employment or self-employed, retired, doing unpaid or voluntary work, or being full or part time students);  1 = Unemployed;  9 = Unknow or missing value. |
| Townsend deprivation index | 22189 | Townsend deprivation index at recruitment | Continuous. | A higher value indicates greater deprivation. |
| Smoking | 20116 | The current/past smoking status of the participant. | Never; Past; Current. | 0 = Never smoker;  1 = Past smoker;  2=Current smoker. |
| Alcohol consumption | 1558 | About how often do you drink alcohol? | Never; Special occasions only; One to three times a month; Once or twice a week; Three or four times a week; Daily or almost daily. | 1 = Never drinking;  2 = Special occasions only;  3 = One to three time a month;  4 = Oner or twice a week;  5 = Three or four times a week;  6 = Daily or almost daily. |
| Adequate physical activity | 884,894,904,914 | Number of days/week of moderate/vigorous physical activity 10+ minutes?  Duration of moderate/vigorous activity? | Continue | Total amount of weekly moderate and vigorous physical activity was calculated by multiplying the number of days per week and the duration per day, respectively. Adequate physical activity was defined as vigorous physical activity ≥75 min/week, or moderate physical activity ≥150min/week, or equivalent combination.  0 = No; 1 = Yes. |
| Diet | **See Table S4** | | | |
| Obesity | 21001 | Body mass index (BMI) | kg/m^2^ | Obesity was defined as BMI ≥30 kg/m^2^, and no obesity was defined as BMI <30 kg/m^2^.  1 = Obesity; 0 = no obesity. |
| Reaction time | 20023 | Mean time to correctly identify matches | Milliseconds | Continue variable |
| Hypertension | 131286-131294 | First occurrences (ICD 10: I10-I15) | Date of first record less than date of baseline recruitment. | 1 = Yes;  0 = No. |
| Diabetes | 130706-130714 | First occurrences (ICD 10: E10-E14) | Date of first record less than date of baseline recruitment. | 1 = Yes;  0 = No |
| Cardiovascular disease | 131296-131306;  131360-131368 | First occurrences (ICD 10: I20-I25 and I60-I64 ) | Date of first record less than date of baseline recruitment. | 1 = Yes;  0 = No |
| Cancer | 40005 | Date of cancer diagnosis | Date of first record less than date of baseline recruitment. | 1 = Yes;  0 = No |

| **Table S4** Measurement of healthy diet used in the UK Biobank | | | |
| --- | --- | --- | --- |
| **Diet component** | **Intake goal** | **Field IDs** | **Amount per serving** |
| Fruit | ≥3 servings/day | 1309 (pieces fresh fruit/day)  1319 (pieces dried fruit/day) | 1309 – 1 piece  1319 – 5 pieces |
| Vegetable | ≥3 servings/day | 1289 (tablespoons cooked vegetables/day)  1299 (salad/raw vegetables/day) | 3 heaped tablespoons |
| Whole grains | ≥3 servings/day | 1438, 1448 (whole meal/wholegrain bread slices/week)  1458, 1468 (bran/oat/muesli cereal bowls/week) | 1438/1448 – 1 slice/day  1458/1468 – 1 bowl/day |
| (Shell)fish | ≥2 servings/week | 1329 (oily fish/week)  1339 (non-oily fish/week) | Once/week |
| Refined grains | ≤2 servings/day | 1438, 1448 (white, brown, other bread slices/week)  1458, 1468 (biscuit, other cereals/week) | 1438/1448 – 1 slice/day  1458/1468 – 1 bowl/day |
| Processed meats | ≤1 serving/week | 1349 (processed meat/week or daily)  3680 (age when last ate meat) | 1349 – 1 piece/day  3680 – 0 pieces/day if indicated having never eaten meat |
| Unprocessed meats | ≤2 servings/week | 1359 (poultry/week or day)  1369 (beef/week or day)  1379 (lamb or mutton/week or day)  1389 (pork/week or day)  3680 (age when last ate meat) | 1359-1389 – once/week  3680 – 0 pieces/day if indicated having never eaten meat |

Field IDs and serving sizes used per diet component in UK Biobank with available data from the general baseline questionnaire. If participants achieved the intake goal, they were considered to have an adequate intake of the diet component.

Scoring criteria for healthy dietary component recommendations: 1: If intake goal met; 0: If intake goal not met (Range: 0-7).

| **Table S5** Baseline characteristics of population according to baseline frailty status | | | | |
| --- | --- | --- | --- | --- |
| **Characteristic** | **Frailty** | | | ***P* value** |
|  | **No frailty** | **Pre-frailty** | **Frailty** |  |
| n | 212828 | 125113 | 8905 |  |
| Age, years (mean [SD]) | 56.92±8.01 | 56.66±8.13 | 57.90±7.72 | <0.001 |
| Townsend deprivation index, (mean [SD]) | -1.75±2.82 | -1.01±3.19 | 0.57±3.56 | <0.001 |
| Healthy diet index, (mean [SD]) | 3.38±1.31 | 3.25±1.33 | 2.95±1.31 | <0.001 |
| Reaction time, ms (mean [SD]) | 553.57±110.16 | 563.72±119.40 | 599.59±145.83 | <0.001 |
| Incidence of delirium |  |  |  | <0.001 |
| No | 209743 (98.55) | 122174 (97.65) | 8375 (94.05) |  |
| Yes | 3085 (1.45) | 2939 (2.35) | 530 (5.95) |  |
| Sex |  |  |  | <0.001 |
| Female | 120089 (56.43) | 67601 (54.03) | 4122 (46.29) |  |
| Male | 92739 (43.57) | 57512 (45.97) | 4783 (53.71) |  |
| Ethnicity |  |  |  | <0.001 |
| White | 196579 (92.37) | 112728 (90.10) | 7813 (87.74) |  |
| Mixed | 6546 (3.08) | 5244 (4.19) | 500 (5.61) |  |
| Asian | 6887 (3.24) | 4262 (3.41) | 278 (3.12) |  |
| Black | 765 (0.36) | 836 (0.67) | 81 (0.91) |  |
| Chinese | 472 (0.22) | 405 (0.32) | 30 (0.34) |  |
| Others | 1579 (0.74) | 1638 (1.31) | 203 (2.28) |  |
| Educational level |  |  |  | <0.001 |
| Less than college | 137476 (64.59) | 89040 (71.17) | 7367 (82.73) |  |
| Equal to or more than college | 73979 (34.76) | 34905 (27.90) | 1420 (15.95) |  |
| Unknow/missing value | 1373 (0.65) | 1168 (0.93) | 118 (1.33) |  |
| Family income |  |  |  | <0.001 |
| <18000 | 33621 (15.80) | 27989 (22.37) | 3822 (42.92) |  |
| 18000 to 51999 | 98975 (46.50) | 56148 (44.88) | 2777 (31.18) |  |
| >52000 | 53726 (25.24) | 23675 (18.92) | 586 (6.58) |  |
| Unknow/missing value | 26506 (12.45) | 17301 (13.83) | 1720 (19.31) |  |
| Employment status |  |  |  | <0.001 |
| Current employed | 124040 (58.28) | 70115 (56.04) | 2551 (28.65) |  |
| Current no employed | 88331 (41.50) | 54586 (43.63) | 6316 (70.93) |  |
| Unknow/missing value | 457 (0.21) | 412 (0.33) | 38 (0.43) |  |
| Smoking status |  |  |  | <0.001 |
| Never smoking | 118486 (55.67) | 63257 (50.56) | 3500 (39.30) |  |
| Ever smoking | 75825 (35.63) | 45275 (36.19) | 3309 (37.16) |  |
| Current smoking | 18025 (8.47) | 16165 (12.92) | 2060 (23.13) |  |
| Unknow/missing value | 492 (0.23) | 416 (0.33) | 36 (0.40) |  |
| Drinking status |  |  |  | <0.001 |
| Never drinking | 12281 (5.77) | 12182 (9.74) | 1835 (20.61) |  |
| Special occasions only | 19487 (9.16) | 17291 (13.82) | 1975 (22.18) |  |
| One to three times a month | 22168 (10.42) | 15235 (12.18) | 1031 (11.58) |  |
| Once or twice a week | 55627 (26.14) | 32552 (26.02) | 1871 (21.01) |  |
| Three or four times a week | 54770 (25.73) | 25063 (20.03) | 1052 (11.81) |  |
| Daily or almost daily | 48441 (22.76) | 22707 (18.15) | 1129 (12.68) |  |
| Unknow/missing value | 54 (0.03) | 83 (0.07) | 12 (0.13) |  |
| Obesity |  |  |  | <0.001 |
| No | 171408 (80.54) | 84554 (67.58) | 4385 (49.24) |  |
| Yes | 41420 (19.46) | 40559 (32.42) | 4520 (50.76) |  |
| Adequate physical activity |  |  |  | <0.001 |
| No | 44098 (20.72) | 40406 (32.30) | 5389 (60.52) |  |
| Yes | 166737 (78.34) | 82026 (65.56) | 2969 (33.34) |  |
| Unknow/missing value | 1993 (0.94) | 2681 (2.14) | 547 (6.14) |  |
| Baseline disease status |  |  |  |  |
| Cardiovascular disease | 12842 (6.03) | 12995 (10.39) | 2358 (26.48) | <0.001 |
| Diabetes | 8069 (3.79) | 11627 (9.29) | 2112 (23.72) | <0.001 |
| Hypertension | 114773 (53.93) | 73469 (58.72) | 6312 (70.88) | <0.001 |
| Dyslipidemia | 98005 (46.05) | 59946 (47.91) | 5169 (58.05) | <0.001 |
| Sleep quality |  |  |  | <0.001 |
| Low | 3162 (1.49) | 4652 (3.72) | 1031 (11.58) |  |
| Intermediate | 76572 (35.98) | 57249 (45.76) | 5220 (58.62) |  |
| High | 133094 (62.54) | 63212 (50.52) | 2654 (29.80) |  |

Data were presented as mean (SD) for continue variables and frequency (%) for categorized variables.

| **Table S6** Baseline characteristics of population according to baseline sleep quality | | | | |
| --- | --- | --- | --- | --- |
| **Characteristic** | **Sleep quality** | | | ***P* value** |
|  | **Low** | **Intermediate** | **High** |  |
| n | 198960 | 139041 | 8845 |  |
| Age, years (mean [SD]) | 56.73±8.18 | 57.04±7.87 | 56.70±7.72 | <0.001 |
| Townsend deprivation index, (mean [SD]) | -1.57±2.93 | -1.26±3.10 | -0.61±3.37 | <0.001 |
| Healthy diet index, (mean [SD]) | 3.41±1.31 | 3.22±1.33 | 3.03±1.35 | <0.001 |
| Reaction time, ms (mean [SD]) | 557.94±114.38 | 558.59±114.89 | 566.17±126.13 | <0.001 |
| Incidence of delirium |  |  |  | <0.001 |
| No | 195569 (98.30) | 136125 (97.90) | 8598 (97.21) |  |
| Yes | 3391 (1.70) | 2916 (2.10) | 247 (2.79) |  |
| Sex |  |  |  | <0.001 |
| Female | 114369 (57.48) | 72877 (52.41) | 4566 (51.62) |  |
| Male | 84591 (42.52) | 66164 (47.59) | 4279 (48.38) |  |
| Ethnicity |  |  |  | <0.001 |
| White | 182308 (91.63) | 126849 (91.23) | 7963 (90.03) |  |
| Mixed | 6754 (3.39) | 5161 (3.71) | 375 (4.24) |  |
| Asian | 6634 (3.33) | 4495 (3.23) | 298 (3.37) |  |
| Black | 911 (0.46) | 710 (0.51) | 61 (0.69) |  |
| Chinese | 520 (0.26) | 373 (0.27) | 14 (0.16) |  |
| Others | 1833 (0.92) | 1453 (1.05) | 134 (1.51) |  |
| Educational level |  |  |  | <0.001 |
| Less than college | 129585 (65.13) | 97597 (70.19) | 6701 (75.76) |  |
| Equal to or more than college | 67890 (34.12) | 40330 (29.01) | 2084 (23.56) |  |
| Unknow/missing value | 1485 (0.75) | 1114 (0.80) | 60 (0.68) |  |
| Family income |  |  |  | <0.001 |
| <18000 | 34442 (17.31) | 28592 (20.56) | 2398 (27.11) |  |
| 18000 to 51999 | 91060 (45.77) | 63121 (45.40) | 3719 (42.05) |  |
| >52000 | 47834 (24.04) | 28733 (20.67) | 1420 (16.05) |  |
| Unknow/missing value | 25624 (12.88) | 18595 (13.37) | 1308 (14.79) |  |
| Employment status |  |  |  | <0.001 |
| Current employed | 115526 (58.06) | 76888 (55.30) | 4292 (48.52) |  |
| Current no employed | 82912 (41.67) | 61787 (44.44) | 4534 (51.26) |  |
| Unknow/missing value | 522 (0.26) | 366 (0.26) | 19 (0.21) |  |
| Smoking status |  |  |  | <0.001 |
| Never smoking | 113386 (56.99) | 68109 (48.98) | 3748 (42.37) |  |
| Ever smoking | 68164 (34.26) | 52750 (37.94) | 3495 (39.51) |  |
| Current smoking | 16906 (8.50) | 17766 (12.78) | 1578 (17.84) |  |
| Unknow/missing value | 504 (0.25) | 416 (0.30) | 24 (0.27) |  |
| Drinking status |  |  |  | <0.001 |
| Never drinking | 14830 (7.45) | 10599 (7.62) | 869 (9.82) |  |
| Special occasions only | 21506 (10.81) | 15972 (11.49) | 1275 (14.41) |  |
| One to three times a month | 21977 (11.05) | 15375 (11.06) | 1082 (12.23) |  |
| Once or twice a week | 52870 (26.57) | 35049 (25.21) | 2131 (24.09) |  |
| Three or four times a week | 47766 (24.01) | 31457 (22.62) | 1662 (18.79) |  |
| Daily or almost daily | 39941 (20.07) | 30514 (21.95) | 1822 (20.60) |  |
| Unknow/missing value | 70 (0.04) | 75 (0.05) | 4 (0.05) |  |
| Obesity |  |  |  | <0.001 |
| No | 158021 (79.42) | 97361 (70.02) | 4965 (56.13) |  |
| Yes | 40939 (20.58) | 41680 (29.98) | 3880 (43.87) |  |
| Adequate physical activity |  |  |  | <0.001 |
| No | 46528 (23.39) | 40100 (28.84) | 3265 (36.91) |  |
| Yes | 149780 (75.28) | 96567 (69.45) | 5385 (60.88) |  |
| Unknow/missing value | 2652 (1.33) | 2374 (1.71) | 195 (2.20) |  |
| Baseline disease status |  |  |  |  |
| Cardiovascular disease | 13825 (6.95) | 13145 (9.45) | 1225 (13.85) | <0.001 |
| Diabetes | 10204 (5.13) | 10477 (7.54) | 1127 (12.74) | <0.001 |
| Hypertension | 106424 (53.49) | 82366 (59.24) | 5764 (65.17) | <0.001 |
| Dyslipidemia | 89273 (44.87) | 68949 (49.59) | 4898 (55.38) | <0.001 |
| Sleep quality |  |  |  | <0.001 |
| Low | 133094 (66.89) | 76572 (55.07) | 3162 (35.75) |  |
| Intermediate | 63212 (31.77) | 57249 (41.17) | 4652 (52.59) |  |
| High | 2654 (1.33) | 5220 (3.75) | 1031 (11.66) |  |

Data were presented as mean (SD) for continue variables and frequency (%) for categorized variables.

| **Table S7** Association between individual components of frailty and risk of delirium | | | | | | | | | | |
| --- | --- | --- | --- | --- | --- | --- | --- | --- | --- | --- |
| **Frailty components** | **Case/*n*** | **Peron-years** | **HR (95% CI)** | | | | | | | |
|  |  |  | **Model 1** | ***P* values** | **Model 2** | ***P* values** | **Model 3** | ***P* values** | **Model 4** | ***P* values** |
| Weight loss | 1175/54013 | 741717 | 1.29 (1.21-1.37) | <0.001 | 1.22 (1.15-1.30) | <0.001 | 1.16 (1.09-1.23) | <0.001 | 1.15 (1.08-1.23) | <0.001 |
| Exhaustion | 1063/43863 | 603175 | 1.79 (1.67-1.91) | <0.001 | 1.43 (1.33-1.53) | <0.001 | 1.31 (1.22-1.41) | <0.001 | 1.22 (1.13-1.30) | <0.001 |
| Slow gait speed | 1378/28029 | 383286 | 2.41 (2.27-2.55) | <0.001 | 1.77 (1.65-1.89) | <0.001 | 1.57 (1.46-1.67) | <0.001 | 1.45 (1.35-1.55) | <0.001 |
| Weaking | 871/18693 | 252525 | 1.63 (1.51-1.76) | <0.001 | 1.39 (1.28-1.50) | <0.001 | 1.28 (1.18-1.38) | <0.001 | 1.21 (1.12-1.31) | <0.001 |
| Low physical activity | 1093/35906 | 494143 | 1.84 (1.72-1.96) | <0.001 | 1.40 (1.30-1.50) | <0.001 | 1.32 (1.23-1.41) | <0.001 | 1.19 (1.10-1.28) | <0.001 |
| Abbreviation: HR hazard ratio; CI confidence interval.  Model 1 adjusted for age and sex; Model 2 additionally adjusted for ethnicity, educational level, family income, TDI, employ status, smoke, drink, obesity, healthy diet score, and physical activity; Model 3 additionally adjusted for disease history of cardiovascular disease, diabetes, hypertension, dyslipidemia, reaction time, and sleep quality. Model 4 mutually adjusted for other frailty components. | | | | | | | | | | |

**Table S8** Association of sleep quality at baseline with risk of incidence delirium

|  | **Case/n** | **Person-years** | **HR (95% CI)** | | | | | |
| --- | --- | --- | --- | --- | --- | --- | --- | --- |
|  |  |  | **Model 1** | ***P* values** | **Model 2** | ***P* values** | **Model 3** | ***P* values** |
| **Sleep quality** |  |  |  |  |  |  |  |  |
| High | 3,391/198,960 | 2,741,271 | 1.00 (Reference) |  | 1.00 (Reference) |  | 1.00 (Reference) |  |
| Intermediate | 2,916/139,041 | 1,913,894 | 1.21 (1.15-1.27) | <0.001 | 1.11 (1.06-1.17) | <0.001 | 1.06 (1.00-1.11) | 0.031 |
| Low | 247/8,845 | 1,215,88 | 1.71 (1.50-1.94) | <0.001 | 1.36 (1.19-1.55) | <0.001 | 1.15 (1.01-1.31) | 0.036 |
| *P* for trend |  |  | <0.001 |  | <0.001 |  | <0.001 |  |
| **Sleep score** |  |  |  |  |  |  |  |  |
| 5 | 1,164/72,514 | 999,274 | 1.00 (Reference) |  | 1.00 (Reference) |  | 1.00 (Reference) |  |
| 4 | 2,227/126,446 | 1,741,997 | 1.05 (0.98-1.13) | 0.141 | 1.02 (0.95-1.10) | 0.543 | 1.01 (0.94-1.09) | 0.71 |
| 3 | 2,001/98,672 | 1,358,510 | 1.20 (1.12-1.29) | <0.001 | 1.11 (1.03-1.19) | 0.007 | 1.08 (1.01-1.16) | 0.033 |
| 2 | 915/40,369 | 555,384 | 1.39 (1.27-1.51) | <0.001 | 1.19 (1.09-1.30) | <0.001 | 1.15 (1.05-1.25) | 0.002 |
| 1 | 220/8,211 | 112,927 | 1.70 (1.47-1.96) | <0.001 | 1.34 (1.16-1.55) | <0.001 | 1.25 (1.08-1.44) | 0.003 |
| 0 | 27/634 | 8,662 | 2.72 (1.85-3.98) | <0.001 | 1.83 (1.25-2.69) | 0.002 | 1.65 (1.12-2.42) | 0.011 |
| Per 1-score decrease |  |  | 1.13 (1.11-1.16) | <0.001 | 1.07 (1.05-1.10) | <0.001 | 1.06 (1.03-1.08) | <0.001 |

Model 1 adjusted for age and sex; Model 2 additionally adjusted for ethnicity, educational level, family income, TDI, employ status, smoke, drink, obesity, healthy diet score, and physical activity; Model 3 additionally adjusted for cardiovascular diseases, diabetes, hypertension, dyslipidemia, reaction time, and frailty status. Abbreviation: HR hazard ratio; CI confidence interval.

| **Table S9** Association between individual sleep behaviors and risk of delirium | | | | | | | | | | |
| --- | --- | --- | --- | --- | --- | --- | --- | --- | --- | --- |
| **Sleep behaviors** | **Case/n** | **Person-years** | **HR (95% CI)** | | | | | | | |
|  |  |  | **Model 1** | ***P* values** | **Model 2** | ***P* values** | **Model 3** | ***P* values** | **Model 4** | ***P* values** |
| Sleep 7-8 h/day | 4,017/234,761 | 3,235,104 | 0.78 (0.74-0.82) | <0.001 | 0.87 (0.83-0.92) | <0.001 | 0.92 (0.88-0.97) | 0.002 | 0.93 (0.88-0.98) | 0.005 |
| Early chronotype | 4,156/217,869 | 2,999,785 | 0.90 (0.86-0.95) | <0.001 | 0.94 (0.89-0.99) | 0.018 | 0.96 (0.92-1.01) | 0.147 | 0.96 (0.91-1.01) | 0.143 |
| No frequent insomnia | 4,355/247,163 | 3,404,994 | 0.84 (0.80-0.88) | <0.001 | 0.90 (0.86-0.95) | <0.001 | 0.96 (0.91-1.01) | 0.159 | 0.98 (0.93-1.04) | 0.522 |
| No snoring | 4,030/216,488 | 2,981,281 | 1.03 (0.98-1.08) | 0.308 | 1.03 (0.97-1.08) | 0.339 | 1.03 (0.98-1.09) | 0.217 | 1.03 (0.98-1.09) | 0.198 |
| No frequent daytime sleepiness | 6,223/337,038 | 4,642,421 | 0.63 (0.57-0.71) | <0.001 | 0.75 (0.67-0.84) | <0.001 | 0.87 (0.78-0.97) | 0.016 | 0.88 (0.78-0.98) | 0.022 |
| Abbreviation: HR hazard ratio; CI confidence interval.  Model 1 adjusted for age and sex; Model 2 additionally adjusted for ethnicity, educational level, family income, TDI, employ status, smoke, drink, obesity, healthy diet score, and physical activity; Model 3 additionally adjusted for disease history of cardiovascular disease, diabetes, hypertension, dyslipidemia, reaction time, and frailty. Model 4 mutually adjusted for other sleep behaviors. | | | | | | | | | | |

| **Table S10** Sensitive analyses for the association of frailty status at baseline with risk of incident delirium after excluding cases within 2 first years of follow-up (n=36) | | | | |
| --- | --- | --- | --- | --- |
|  | **Case/n** | **HR (95% CI)** | | |
|  |  | **Model 1** | **Model 2** | **Model 3** |
| **Frailty** |  |  |  |  |
| Non-frailty | 3074/212817 | Reference | Reference | Reference |
| Pre-frailty | 2920/125094 | 1.63 (1.55-1.72) | 1.42 (1.35-1.50) | 1.34 (1.27-1.41) |
| Frailty | 524/8899 | 3.64 (3.32-4.00) | 2.36 (2.14-2.61) | 1.96 (1.77-2.17) |
| **Frailty score** |  |  |  |  |
| 0 | 3074/212817 | Reference | Reference | Reference |
| 1 | 2007/98168 | 1.45 (1.37-1.53) | 1.31 (1.24-1.39) | 1.26 (1.19-1.34) |
| 2 | 913/26926 | 2.27 (2.11-2.45) | 1.78 (1.65-1.93) | 1.60 (1.48-1.73) |
| 3 | 407/7287 | 3.50 (3.16-3.89) | 2.38 (2.13-2.66) | 2.00 (1.78-2.23) |
| 4 | 103/1481 | 4.13 (3.39-5.03) | 2.57 (2.10-3.15) | 2.04 (1.66-2.50) |
| 5 | 14/131 | 5.37 (3.17-9.07) | 3.19 (1.88-5.42) | 2.61 (1.54-4.43) |
| Per 1-score increase | 6518/346810 | 1.49 (1.45-1.52) | 1.32 (1.28-1.35) | 1.24 (1.21-1.28) |
| Model 1 adjusted for age and sex; Model 2 additionally adjusted for ethnicity, educational level, family income, TDI, employ status, smoke, drink, obesity, healthy diet score, and physical activity; Model 3 additionally adjusted for cardiovascular diseases, diabetes, hypertension, dyslipidemia, reaction time, and sleep quality. Abbreviation: HR hazard ratio; CI confidence interval. | | | | |

| **Table S11** Sensitive analyses for the association of frailty status at baseline with risk of incident delirium after imputing missing data on covariates | | | | |
| --- | --- | --- | --- | --- |
|  | **Case/n** | **HR (95% CI)** | | |
|  |  | **Model 1** | **Model 2** | **Model 3** |
| **Frailty** |  |  |  |  |
| Non-frailty | 3085/212828 | Reference | Reference | Reference |
| Pre-frailty | 2939/125113 | 1.64 (1.56-1.72) | 1.42 (1.35-1.50) | 1.34 (1.27-1.41) |
| Frailty | 530/8905 | 3.67 (3.35-4.03) | 2.39 (2.16-2.64) | 1.98 (1.78-2.19) |
| **Frailty score** |  |  |  |  |
| 0 | 3085/212828 | Reference | Reference | Reference |
| 1 | 2020/98181 | 1.45 (1.37-1.54) | 1.32 (1.25-1.40) | 1.26 (1.19-1.34) |
| 2 | 919/26932 | 2.28 (2.12-2.45) | 1.79 (1.66-1.94) | 1.60 (1.48-1.74) |
| 3 | 412/7292 | 3.54 (3.19-3.92) | 2.41 (2.16-2.69) | 2.02 (1.80-2.26) |
| 4 | 104/1482 | 4.15 (3.41-5.05) | 2.59 (2.11-3.16) | 2.05 (1.67-2.52) |
| 5 | 14/131 | 5.34 (3.16-9.04) | 3.18 (1.88-5.40) | 2.60 (1.53-4.42) |
| Per 1-score increase | 6554/346846 | 1.49 (1.45-1.52) | 1.32 (1.28-1.35) | 1.25 (1.21-1.28) |
| Model 1 adjusted for age and sex; Model 2 additionally adjusted for ethnicity, educational level, family income, TDI, employ status, smoke, drink, obesity, healthy diet score, and physical activity; Model 3 additionally adjusted for cardiovascular diseases, diabetes, hypertension, dyslipidemia, reaction time, and sleep quality. Abbreviation: HR hazard ratio; CI confidence interval. | | | | |

| **Table S12** Sensitive analyses for the association of frailty status at baseline with risk of incident delirium after excluding cases of dementia-related delirium (n=1260) | | | | |
| --- | --- | --- | --- | --- |
|  | **Case/n** | **HR (95% CI)** | | |
|  |  | **Model 1** | **Model 2** | **Model 3** |
| **Frailty** |  |  |  |  |
| Non-frailty | 2426/212169 | Reference | Reference | Reference |
| Pre-frailty | 2415/124589 | 1.71 (1.62-1.81) | 1.47 (1.38-1.56) | 1.38 (1.30-1.47) |
| Frailty | 453/8828 | 4.01 (3.63-4.44) | 2.51 (2.25-2.80) | 2.08 (1.86-2.33) |
| **Frailty score** |  |  |  |  |
| 0 | 2426/212169 | Reference | Reference | Reference |
| 1 | 1662/97823 | 1.52 (1.43-1.62) | 1.36 (1.28-1.45) | 1.31 (1.23-1.40) |
| 2 | 753/26766 | 2.39 (2.20-2.59) | 1.83 (1.68-1.99) | 1.64 (1.50-1.79) |
| 3 | 354/7234 | 3.89 (3.48-4.35) | 2.54 (2.26-2.87) | 2.14 (1.89-2.42) |
| 4 | 86/1464 | 4.38 (3.53-5.44) | 2.61 (2.09-3.26) | 2.08 (1.66-2.60) |
| 5 | 13/130 | 6.27 (3.63-10.82) | 3.59 (2.07-6.21) | 2.90 (1.68-5.04) |
| Per 1-score increase | 5294/345586 | 1.53 (1.49-1.57) | 1.34 (1.30-1.38) | 1.26 (1.23-1.30) |
| Model 1 adjusted for age and sex; Model 2 additionally adjusted for ethnicity, educational level, family income, TDI, employ status, smoke, drink, obesity, healthy diet score, and physical activity; Model 3 additionally adjusted for cardiovascular diseases, diabetes, hypertension, dyslipidemia, reaction time, and sleep quality. Abbreviation: HR hazard ratio; CI confidence interval. | | | | |

| **Table S13** Sensitive analyses for the association of frailty status at baseline with risk of incident delirium using postoperative delirium as outcome (n=3838) | | | | |
| --- | --- | --- | --- | --- |
|  | **Case/n** | **HR (95% CI)** | | |
|  |  | **Model 1** | **Model 2** | **Model 3** |
| **Frailty** |  |  |  |  |
| Non-frailty | 1909/212828 | Reference | Reference | Reference |
| Pre-frailty | 1685/125113 | 1.51 (1.42-1.61) | 1.35 (1.26-1.45) | 1.29 (1.21-1.38) |
| Frailty | 244/8905 | 2.72 (2.38-3.11) | 1.92 (1.67-2.22) | 1.67 (1.44-1.93) |
| **Frailty score** |  |  |  |  |
| 0 | 1909/212828 | Reference | Reference | Reference |
| 1 | 1177/98181 | 1.36 (1.27-1.47) | 1.26 (1.17-1.36) | 1.22 (1.14-1.32) |
| 2 | 508/26932 | 2.03 (1.84-2.24) | 1.68 (1.51-1.86) | 1.54 (1.39-1.71) |
| 3 | 189/7292 | 2.61 (2.25-3.04) | 1.92 (1.64-2.25) | 1.68 (1.43-1.98) |
| 4 | 51/1482 | 3.24 (2.45-4.28) | 2.23 (1.67-2.96) | 1.87 (1.40-2.49) |
| 5 | 4/131 | 2.39 (0.90-6.38) | 1.60 (0.60-4.29) | 1.38 (0.52-3.71) |
| Per 1-score increase | 3838/346846 | 1.38 (1.33-1.42) | 1.25 (1.21-1.30) | 1.20 (1.16-1.25) |
| Model 1 adjusted for age and sex; Model 2 additionally adjusted for ethnicity, educational level, family income, TDI, employ status, smoke, drink, obesity, healthy diet score, and physical activity; Model 3 additionally adjusted for cardiovascular diseases, diabetes, hypertension, dyslipidemia, reaction time, and sleep quality. Abbreviation: HR hazard ratio; CI confidence interval. | | | | |

| **Table S14** Sensitivity analyses of the association between frailty and sleep quality and risk of delirium: excluding individuals with missing data on covariates (n=292,713) | | | |
| --- | --- | --- | --- |
| **Exposure** | **Hazard ratios (95 confidence interval)** | | |
|  | **Model 1** | **Model 2** | **Model 3** |
| **Individual association** |  |  |  |
| Frailty |  |  |  |
| Non-frailty | 1.00 (Reference) | 1.00 (Reference) | 1.00 (Reference) |
| Pre-frailty | 1.63 (1.54-1.72) | 1.63 (1.54-1.72) | 1.63 (1.54-1.72) |
| Frailty | 3.72 (3.33-4.15) | 3.72 (3.33-4.15) | 3.72 (3.33-4.15) |
| Sleep quality |  |  |  |
| High | 1.00 (Reference) | 1.00 (Reference) | 1.00 (Reference) |
| Intermediate | 1.22 (1.15-1.29) | 1.12 (1.06-1.19) | 1.07 (1.01-1.13) |
| Low | 1.70 (1.46-1.97) | 1.36 (1.17-1.58) | 1.15 (0.99-1.33) |
| **Joint association** |  |  |  |
| Healthy sleep |  |  |  |
| Non-frailty | 1.00 (Reference) | 1.00 (Reference) | 1.00 (Reference) |
| Pre-frailty | 1.60 (1.39-1.84) | 1.42 (1.23-1.63) | 1.35 (1.18-1.56) |
| Frailty | 2.24 (1.36-3.68) | 1.54 (0.94-2.54) | 1.29 (0.78-2.13) |
| Poor sleep |  |  |  |
| Non-frailty | 1.13 (1.02-1.24) | 1.09 (0.99-1.20) | 1.08 (0.98-1.19) |
| Pre-frailty | 1.82 (1.65-2.01) | 1.54 (1.39-1.70) | 1.44 (1.30-1.59) |
| Frailty | 4.24 (3.70-4.85) | 2.67 (2.31-3.08) | 2.21 (1.91-2.56) |
| Model 1 adjusted for age and sex; Model 2 additionally adjusted for ethnicity, educational level, family income, TDI, employ status, smoke, drink, obesity, healthy diet score, and physical activity; Model 3 additionally adjusted for cardiovascular diseases, diabetes, hypertension, dyslipidemia, reaction time, and sleep score or frailty. | | | |

| **Table S15** Sensitivity analyses of the association between frailty and sleep quality and risk of delirium: additionally adjusting for cancer at baseline | | | | | |
| --- | --- | --- | --- | --- | --- |
| **Exposure** | **Hazard ratios (95 confidence interval)** | | |  |  |
|  | **Model 1** | **Model 2** | **Model 3** |  |  |
| **Individual association** |  |  |  |  |  |
| Frailty |  |  |  |  |  |
| Non-frailty | 1.00 (Reference) | 1.00 (Reference) | 1.00 (Reference) |  |  |
| Pre-frailty | 1.64 (1.56-1.72) | 1.42 (1.35-1.50) | 1.34 (1.27-1.41) |  |  |
| Frailty | 3.67 (3.35-4.03) | 2.38 (2.15-2.63) | 1.97 (1.78-2.19) |  |  |
| Sleep quality |  |  |  |  |  |
| High | 1.00 (Reference) | 1.00 (Reference) | 1.00 (Reference) |  |  |
| Intermediate | 1.21 (1.15-1.27) | 1.11 (1.06-1.17) | 1.06 (1.00-1.11) |  |  |
| Low | 1.71 (1.50-1.94) | 1.36 (1.19-1.55) | 1.15 (1.01-1.31) |  |  |
| **Joint association** |  |  |  |  |  |
| Healthy sleep |  |  |  |  |  |
| Non-frailty | 1.00 (Reference) | 1.00 (Reference) | 1.00 (Reference) |  |  |
| Pre-frailty | 1.56 (1.39-1.76) | 1.37 (1.22-1.55) | 1.32 (1.17-1.48) |  |  |
| Frailty | 2.20 (1.45-3.33) | 1.48 (0.97-2.24) | 1.27 (0.84-1.93) |  |  |
| Poor sleep |  |  |  |  |  |
| Non-frailty | 1.04 (0.96-1.14) | 1.01 (0.93-1.10) | 1.00 (0.92-1.09) |  |  |
| Pre-frailty | 1.72 (1.58-1.87) | 1.45 (1.33-1.58) | 1.36 (1.25-1.48) |  |  |
| Frailty | 3.93 (3.50-4.41) | 2.48 (2.19-2.80) | 2.07 (1.83-2.35) |  |  |
| Model 1 adjusted for age and sex; Model 2 additionally adjusted for ethnicity, educational level, family income, TDI, employ status, smoke, drink, obesity, healthy diet score, and physical activity; Model 3 additionally adjusted for cardiovascular diseases, diabetes, hypertension, dyslipidemia, cancer, reaction time, and sleep score or frailty. | | | |  |  |

| **Table S16** Stratified analyses by the follow-up period (≤5 years vs >5 years) of the association between frailty and sleep quality and risk of delirium: excluding individuals with cardiovascular disease at baseline | | |
| --- | --- | --- |
| **Exposure** | **Hazard ratios (95 confidence interval)** | |
|  | **Incident delirium the first 5 years of follow-up** | **Incident delirium after the first 5 years of follow-up** |
| Non-frailty | 1.00 (Reference) | 1.00 (Reference) |
| Pre-frailty | 1.28 (0.95-1.73) | 1.33 (1.26-1.40) |
| Frailty | 1.76 (1.02-3.04) | 1.95 (1.75-2.17) |
| Model 1 adjusted for age and sex; Model 2 additionally adjusted for ethnicity, educational level, family income, TDI, employ status, smoke, drink, obesity, healthy diet score, and physical activity; Model 3 additionally adjusted for cardiovascular diseases, diabetes, hypertension, dyslipidemia, reaction time, and sleep score or frailty. | | |

| **Table S17** Sensitivity analyses of the association between frailty and sleep quality and risk of delirium: excluding individuals with cardiovascular disease at baseline (n=2,8195) | | | | | |  |  |
| --- | --- | --- | --- | --- | --- | --- | --- |
| **Exposure** | **Hazard ratios (95 confidence interval)** | | |  |  |  |  |
|  | **Model 1** | **Model 2** | **Model 3** |  |  |  |  |
| **Individual association** |  |  |  |  |  |  |  |
| Frailty |  |  |  |  |  |  |  |
| Non-frailty | 1.00 (Reference) | 1.00 (Reference) | 1.00 (Reference) |  |  |  |  |
| Pre-frailty | 1.61 (1.52-1.70) | 1.41 (1.33-1.50) | 1.35 (1.28-1.44) |  |  |  |  |
| Frailty | 3.90 (3.48-4.36) | 2.56 (2.27-2.89) | 2.27 (2.01-2.57) |  |  |  |  |
| Sleep quality |  |  |  |  |  |  |  |
| High | 1.00 (Reference) | 1.00 (Reference) | 1.00 (Reference) |  |  |  |  |
| Intermediate | 1.17 (1.11-1.24) | 1.08 (1.03-1.15) | 1.03 (0.98-1.09) |  |  |  |  |
| Low | 1.64 (1.41-1.91) | 1.34 (1.15-1.56) | 1.14 (0.98-1.33) |  |  |  |  |
| **Joint association** |  |  |  |  |  |  |  |
| Healthy sleep |  |  |  |  |  |  |  |
| Non-frailty | 1.00 (Reference) | 1.00 (Reference) | 1.00 (Reference) |  |  |  |  |
| Pre-frailty | 1.62 (1.43-1.85) | 1.44 (1.27-1.64) | 1.40 (1.23-1.59) |  |  |  |  |
| Frailty | 2.19 (1.31-3.66) | 1.48 (0.88-2.47) | 1.32 (0.79-2.21) |  |  |  |  |
| Poor sleep |  |  |  |  |  |  |  |
| Non-frailty | 1.04 (0.95-1.14) | 1.01 (0.92-1.10) | 1.00 (0.91-1.10) |  |  |  |  |
| Pre-frailty | 1.66 (1.52-1.83) | 1.41 (1.29-1.55) | 1.35 (1.23-1.49) |  |  |  |  |
| Frailty | 4.17 (3.64-4.78) | 2.67 (2.31-3.08) | 2.39 (2.07-2.76) |  |  |  |  |
| Model 1 adjusted for age and sex; Model 2 additionally adjusted for ethnicity, educational level, family income, TDI, employ status, smoke, drink, obesity, healthy diet score, and physical activity; Model 3 additionally adjusted for cardiovascular diseases, diabetes, hypertension, dyslipidemia, reaction time, and sleep score or frailty. | | | | | | | |

| **Table S18** Sensitivity analyses of the association between frailty and sleep quality and risk of delirium: excluding individuals who occurred delirium and all-cause dementia within the first 2 years of follow-up (n=187) | | | | | |  |  |
| --- | --- | --- | --- | --- | --- | --- | --- |
| **Exposure** | **Hazard ratios (95 confidence interval)** | | |  |  |  |  |
|  | **Model 1** | **Model 2** | **Model 3** |  |  |  |  |
| **Individual association** |  |  |  |  |  |  |  |
| Frailty |  |  |  |  |  |  |  |
| Non-frailty | 1.00 (Reference) | 1.00 (Reference) | 1.00 (Reference) |  |  |  |  |
| Pre-frailty | 1.63 (1.55-1.72) | 1.42 (1.35-1.50) | 1.34 (1.27-1.41) |  |  |  |  |
| Frailty | 3.65 (3.33-4.01) | 2.37 (2.14-2.62) | 1.96 (1.77-2.18) |  |  |  |  |
| Sleep quality |  |  |  |  |  |  |  |
| High | 1.00 (Reference) | 1.00 (Reference) | 1.00 (Reference) |  |  |  |  |
| Intermediate | 1.21 (1.15-1.27) | 1.11 (1.06-1.17) | 1.06 (1.01-1.11) |  |  |  |  |
| Low | 1.71 (1.50-1.95) | 1.36 (1.19-1.55) | 1.15 (1.01-1.32) |  |  |  |  |
| **Joint association** |  |  |  |  |  |  |  |
| Healthy sleep |  |  |  |  |  |  |  |
| Non-frailty | 1.00 (Reference) | 1.00 (Reference) | 1.00 (Reference) |  |  |  |  |
| Pre-frailty | 1.56 (1.39-1.76) | 1.38 (1.22-1.55) | 1.32 (1.17-1.49) |  |  |  |  |
| Frailty | 2.21 (1.46-3.35) | 1.49 (0.98-2.26) | 1.28 (0.84-1.95) |  |  |  |  |
| Poor sleep |  |  |  |  |  |  |  |
| Non-frailty | 1.04 (0.96-1.14) | 1.01 (0.93-1.10) | 1.01 (0.92-1.09) |  |  |  |  |
| Pre-frailty | 1.71 (1.57-1.87) | 1.44 (1.32-1.57) | 1.36 (1.24-1.48) |  |  |  |  |
| Frailty | 3.90 (3.48-4.38) | 2.46 (2.17-2.78) | 2.06 (1.82-2.34) |  |  |  |  |
| Model 1 adjusted for age and sex; Model 2 additionally adjusted for ethnicity, educational level, family income, TDI, employ status, smoke, drink, obesity, healthy diet score, and physical activity; Model 3 additionally adjusted for cardiovascular diseases, diabetes, hypertension, dyslipidemia, reaction time, and sleep score or frailty. | | | | | | | |

**Table S19** Subgroup analyses of the association between number of frailty components and risk of delirium according to potential effect modifiers

| **Subgroups** | **Per 1-score increase in frailty score** | ***P* for interaction*** |
| --- | --- | --- |
| **Age** |  | 0.005 |
| < 60 years | 1.20 (1.12-1.28) |  |
| ≥ 60 years | 1.24 (1.20-1.28) |  |
| **Sex** |  | <0.001 |
| Female | 1.32 (1.26-1.38) |  |
| Male | 1.21 (1.17-1.25) |  |
| **Obesity** |  | 0.317 |
| No | 1.26 (1.22-1.31) |  |
| Yes | 1.23 (1.17-1.28) |  |
| **Drinking** |  | 0.274 |
| ≤2 times/week | 1.24 (1.20-1.28) |  |
| ≥ 3 times/week | 1.28 (1.22-1.34) |  |
| **Smoking** |  | 0.126 |
| Never | 1.22 (1.17-1.28) |  |
| Ever/current | 1.16 (1.09-1.24) |  |
| **Education** |  | 0.108 |
| Less | 1.25 (1.21-1.29) |  |
| College/university | 1.27 (1.18-1.35) |  |
| **Townsend deprivation index (TDI)** |  | 0.875 |
| Greater deprivation (TDI ≥ median value) | 1.24 (1.20-1.29) |  |
| Less deprivation (TDI < median value) | 1.26 (1.21-1.32) |  |

Abbreviation: CVD cardiovascular disease.

*Interaction was examined using the likelihood ratio test comparing the model with and without an interaction between the number of frailty components and stratified variables.

All models were adjusted for age, sex, ethnicity, educational level, family income, Townsend Deprivation Index, employment status, smoking status, drinking status, obesity, healthy diet scores, physical activity, cardiovascular diseases, diabetes, hypertension, dyslipidemia, reaction time, and sleep quality.


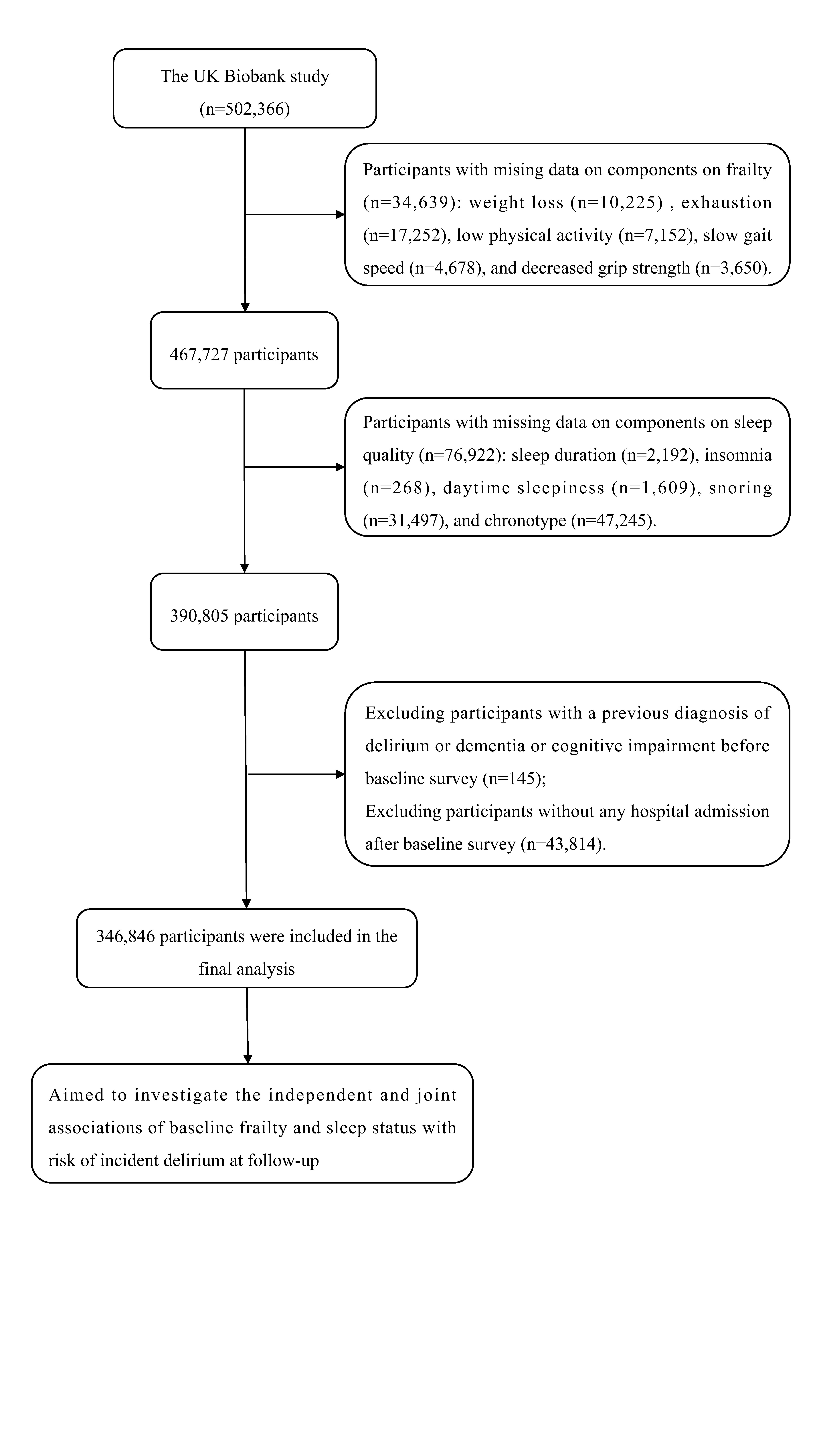


**Figure S1** Flow chart for study participant selection

**A.**

**
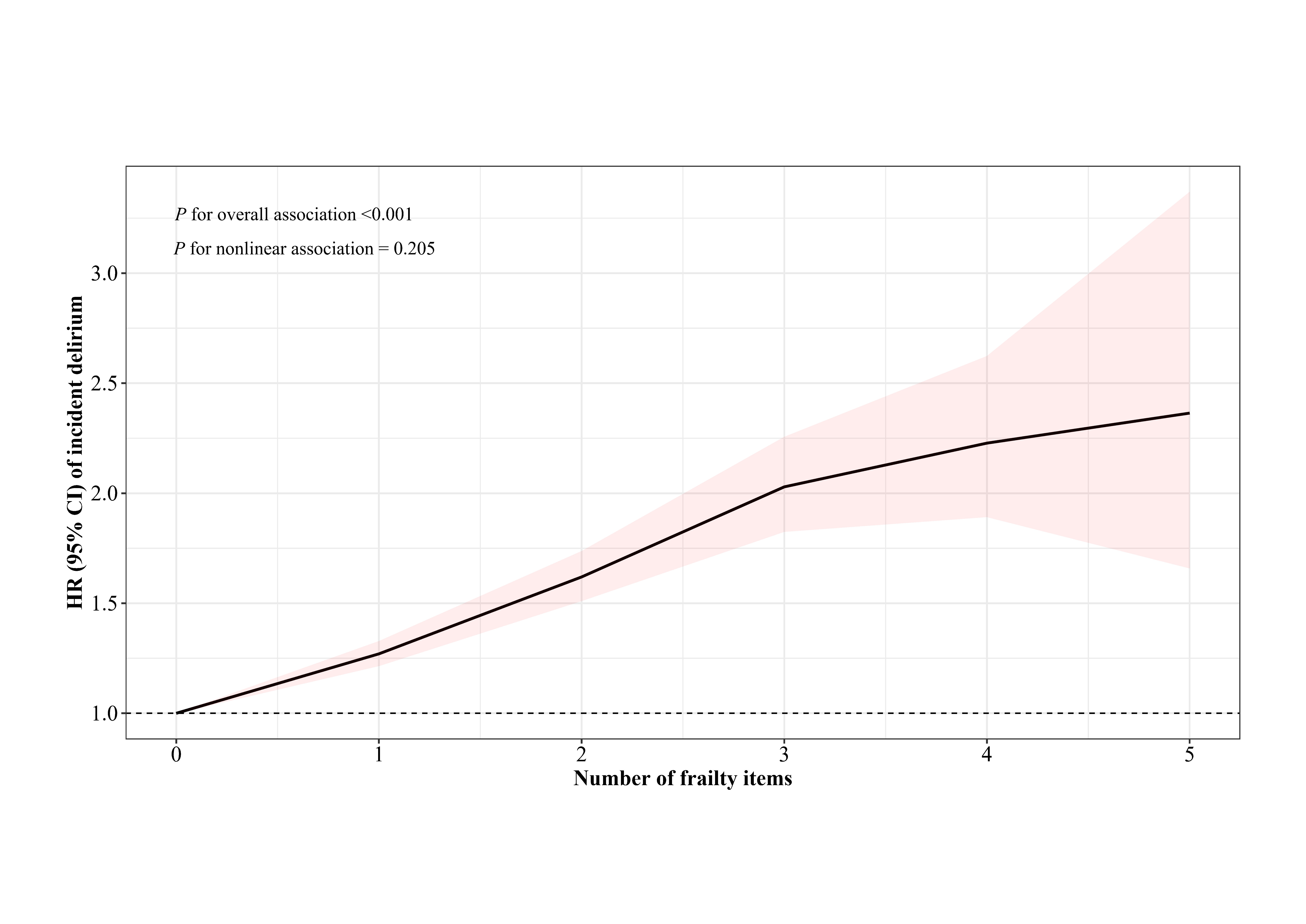
**

**
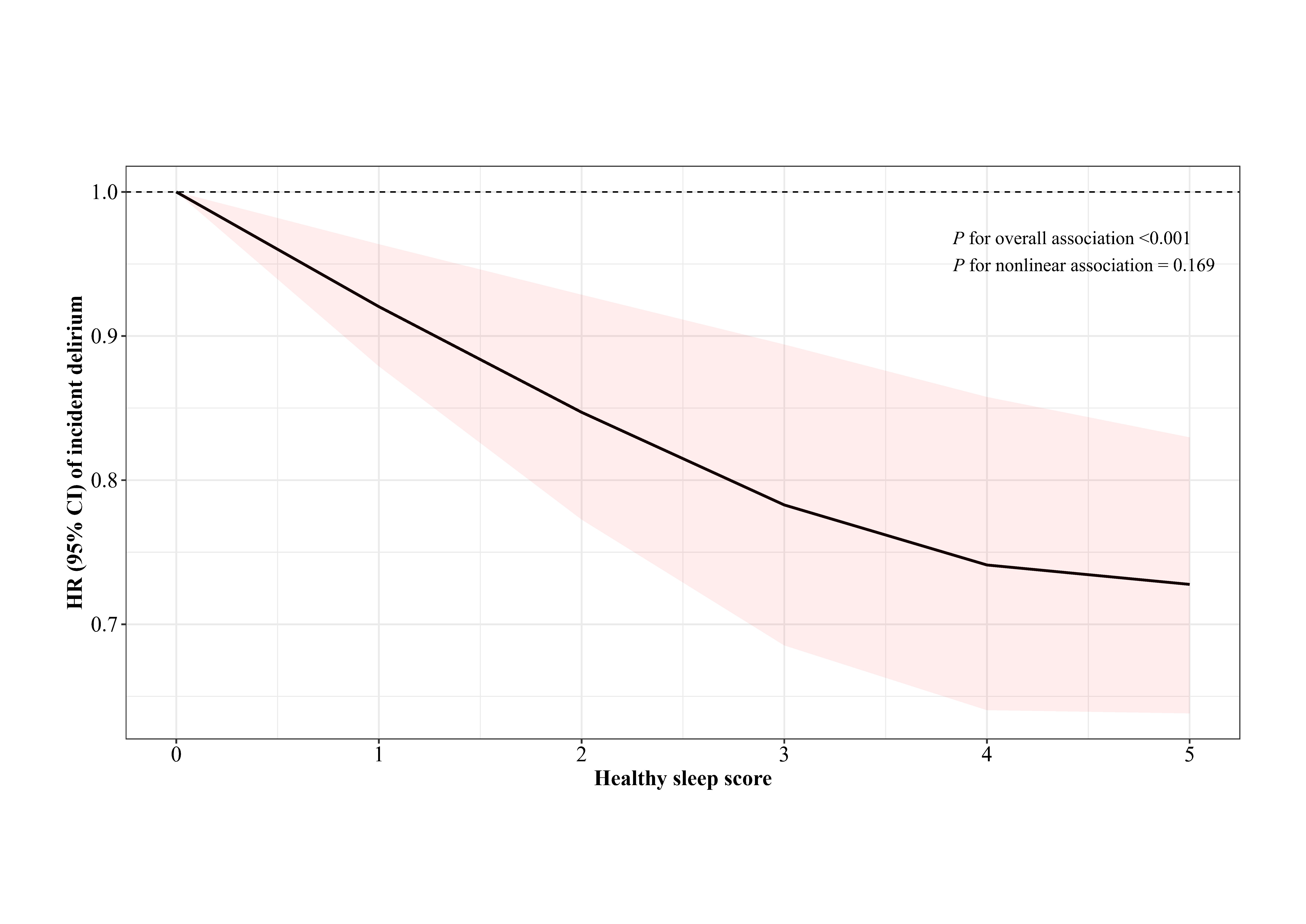
B.**

**Figure S2** Dose-response relationship of frailty score (A) and sleep score (B) with risk of incident delirium

All models were adjusted for age, sex, ethnicity, educational level, family income, Townsend Deprivation Index, employment status, smoking status, drinking status, obesity, healthy diet scores, physical activity, cardiovascular diseases, diabetes, hypertension, dyslipidemia, and reaction time.
